# Supplementary figures and images for: Mechanism underlying the DNA-binding preferences of the Vibrio cholerae and vibriophage VP882 VqmA quorum-sensing receptors
Source: PLoS Genet. 2021 Jul 6;17(7):e1009550. doi: 10.1371/journal.pgen.1009550 (PMC8284805; doi:10.1371/journal.pgen.1009550)

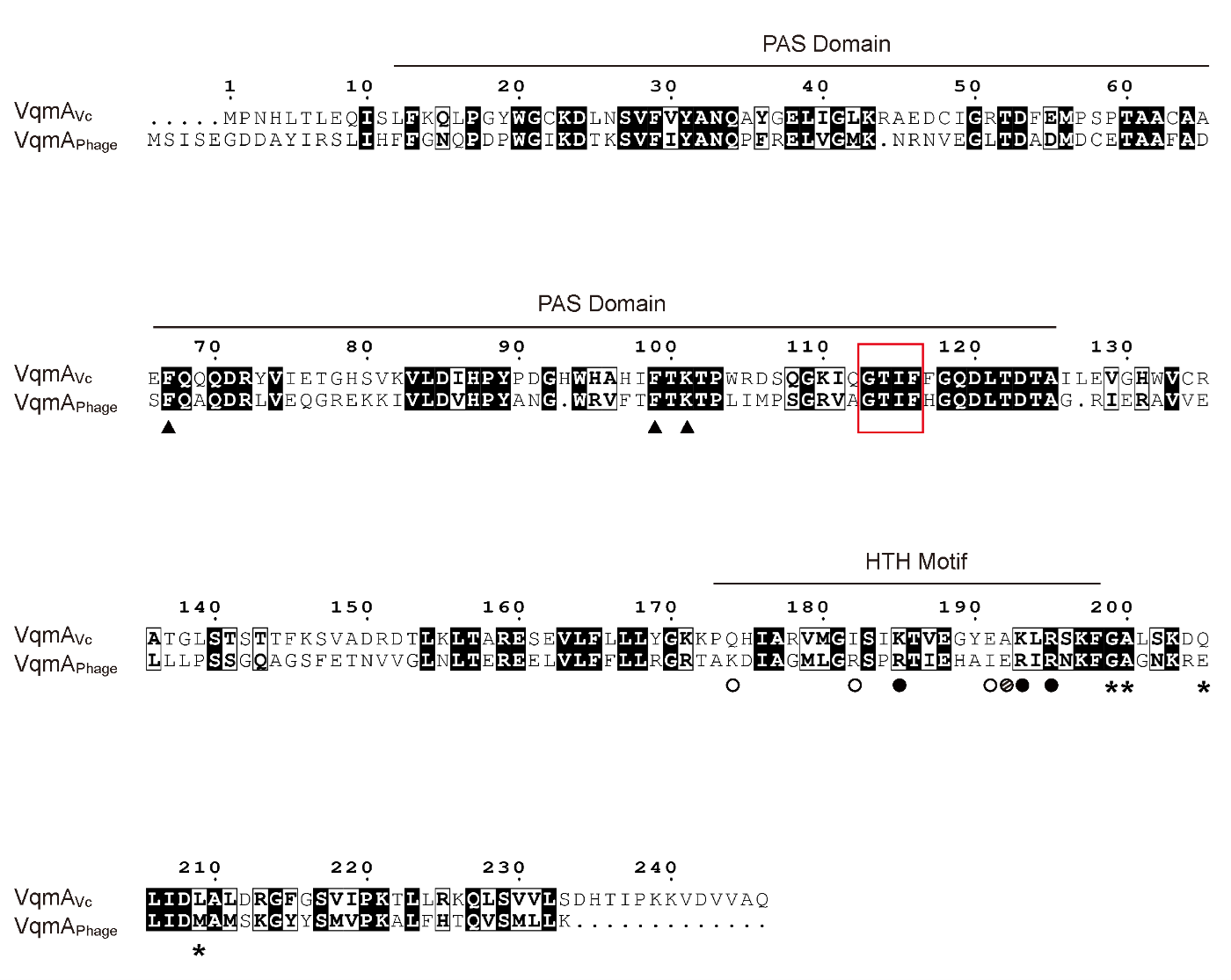

Supplement: S1 Fig — Protein sequence alignment (ClustalW) showing VqmAVc and VqmAPhage. Black and white boxes designate identical and conserved residues, respectively. The PAS domain and HTH motif are indicated. The site used to fuse domains for chimera constructions is indicated by the red box. Key residues required for DPO binding are designated with black triangles. Conserved HTH residues are designated by black circles and open circles show residues with different charges in the HTH motifs of the two proteins. The residue in each HTH motif that contributes to Pqtip specificity is designated by the striped circle. The residues identified in the VqmAPhage screen and the equivalent residues altered by site-directed mutagenesis in VqmAVc are designated by asterisks. (TIF) [file pgen.1009550.s001.tif]

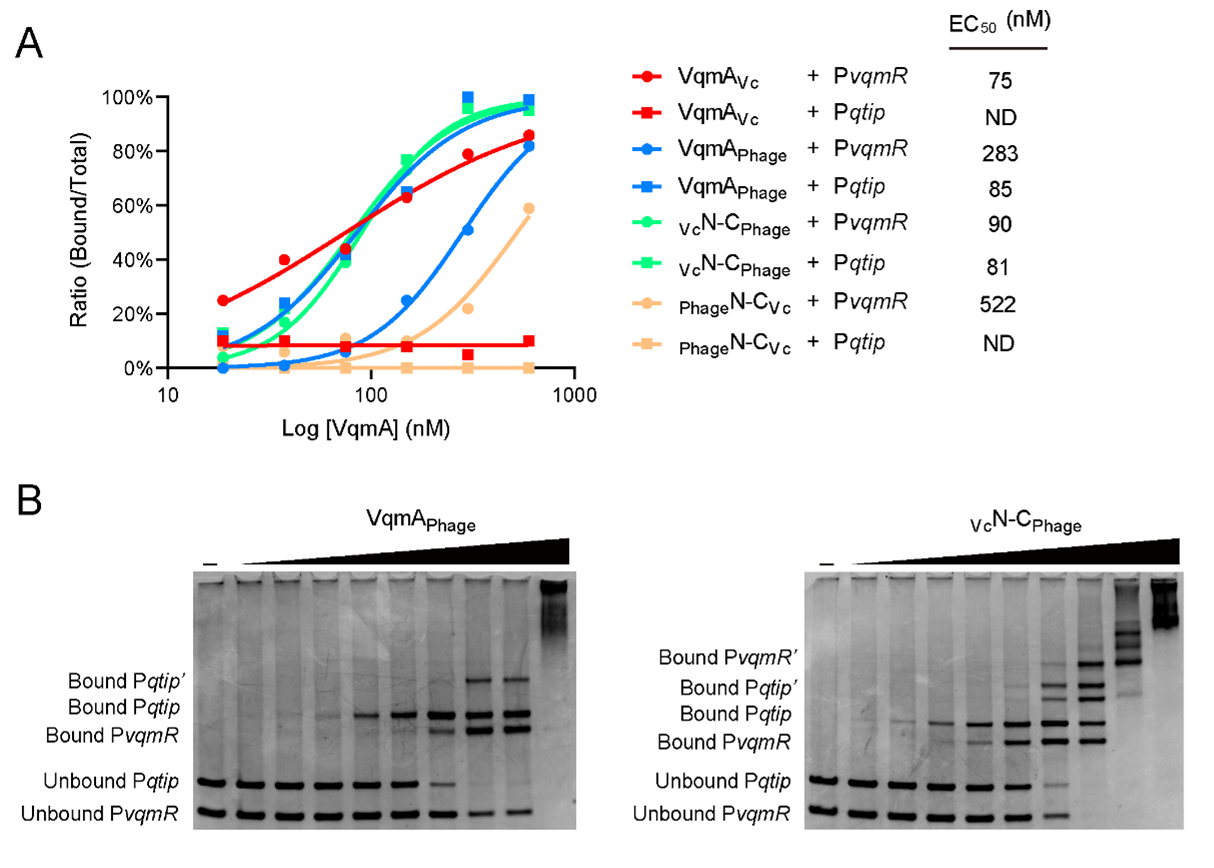

Supplement: S2 Fig — (A) EC50 analysis of the designated VqmA proteins for binding to PvqmR and Pqtip. Data are representative of two independent experiments. The percentage of DNA bound was calculated using the gel analyzer tool in ImageJ and the estimated EC50 values were derived from Prism. (B) Competitive VqmAPhage and VcN-CPhage EMSA analysis. 25 nM PvqmR and Pqtip DNA were used and no protein (designated -) or 2-fold serially-diluted protein was added to the lanes. The lowest and highest protein (dimer) concentrations are 4.7 nM and 1200 nM, respectively. (TIF) [file pgen.1009550.s002.tif]

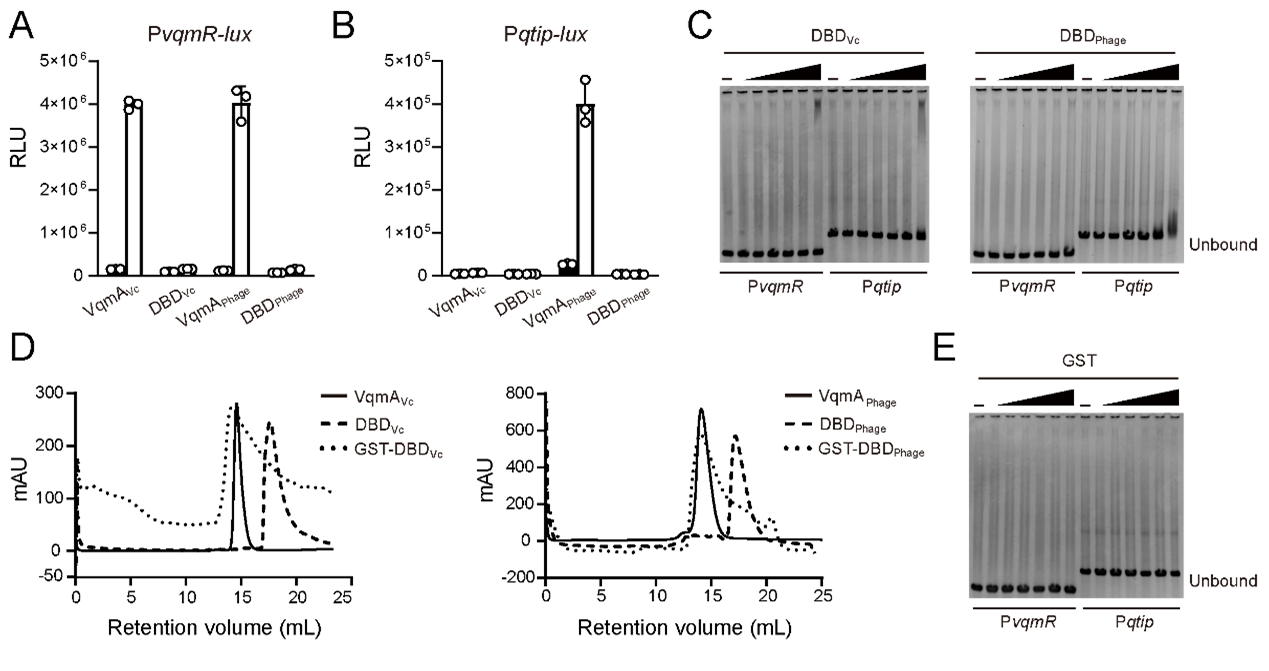

Supplement: S3 Fig — (A and B) Normalized reporter activity from WT E. coli harboring (A) PvqmR-lux or (B) Pqtip-lux and arabinose-inducible VqmAVc, DBDVc, VqmAPhage, and DBDPhage. Black, no arabinose; white, 0.2% arabinose. Data are represented as mean ± SD (error bars) with n = 3 biological replicates. (C) EMSAs of DBDVc and DBDPhage proteins binding to PvqmR and Pqtip. 25 nM PvqmR or Pqtip DNA was used in all EMSAs with no protein (designated -) or 2-fold serial dilutions of proteins. The lowest and highest protein (dimer) concentrations are 18.75 nM and 600 nM, respectively. (D) Gel filtration chromatogram showing UV280 traces for the purification of (left) VqmAVc, DBDVc, and GST-DBDVc and (right) VqmAPhage, DBDPhage, and GST-DBDPhage proteins. (E) EMSA of GST protein binding to PvqmR and Pqtip DNA as in panel C. (TIF) [file pgen.1009550.s003.tif]

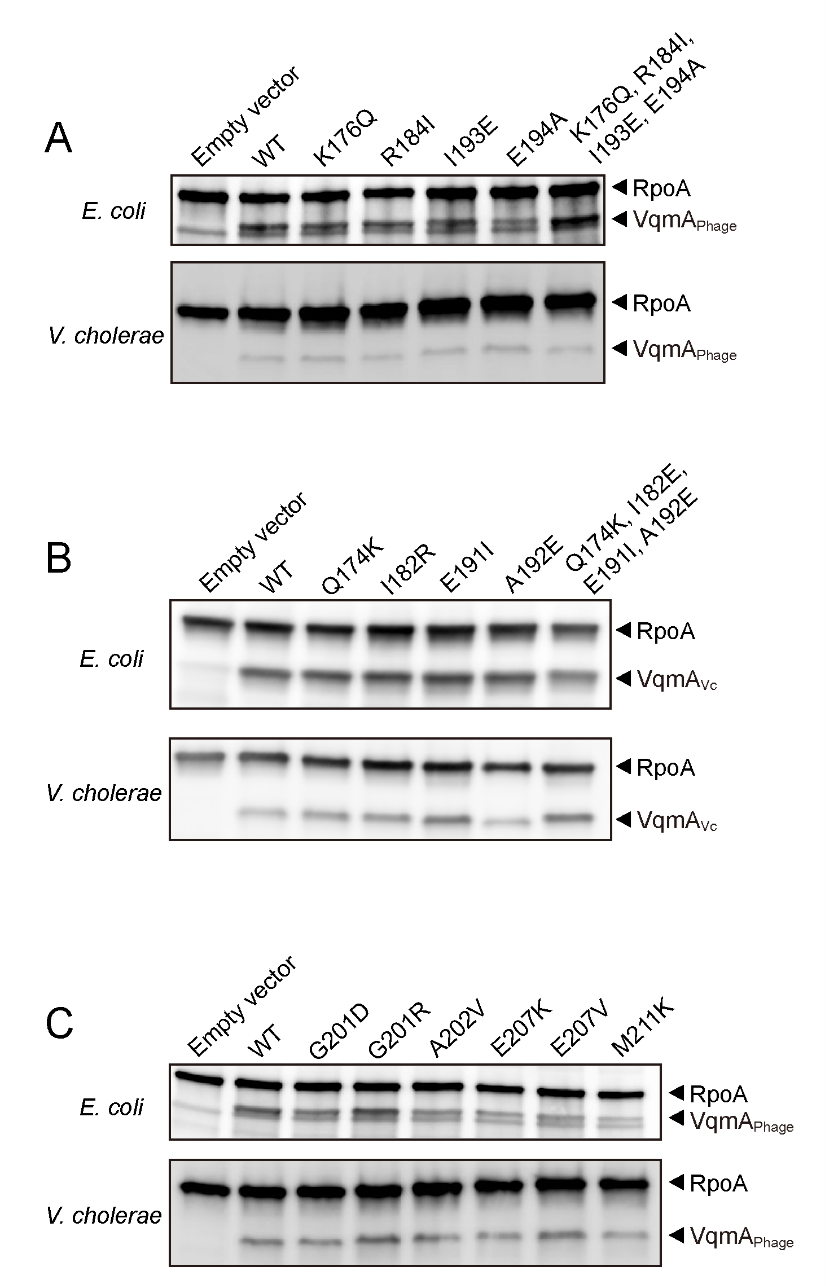

Supplement: S4 Fig — Western blot showing the designated (A and C) 3xFLAG-VqmAPhage and (B) 3xFLAG-VqmAVc proteins produced by Δtdh E. coli and Δtdh ΔvqmAVc V. cholerae. A contaminating band below VqmAPhage and VqmAVc is present in all Δtdh E. coli samples. The RNAPα subunit (RpoA) was used as the loading control. Data are representative of two independent experiments. (TIF) [file pgen.1009550.s004.tif]

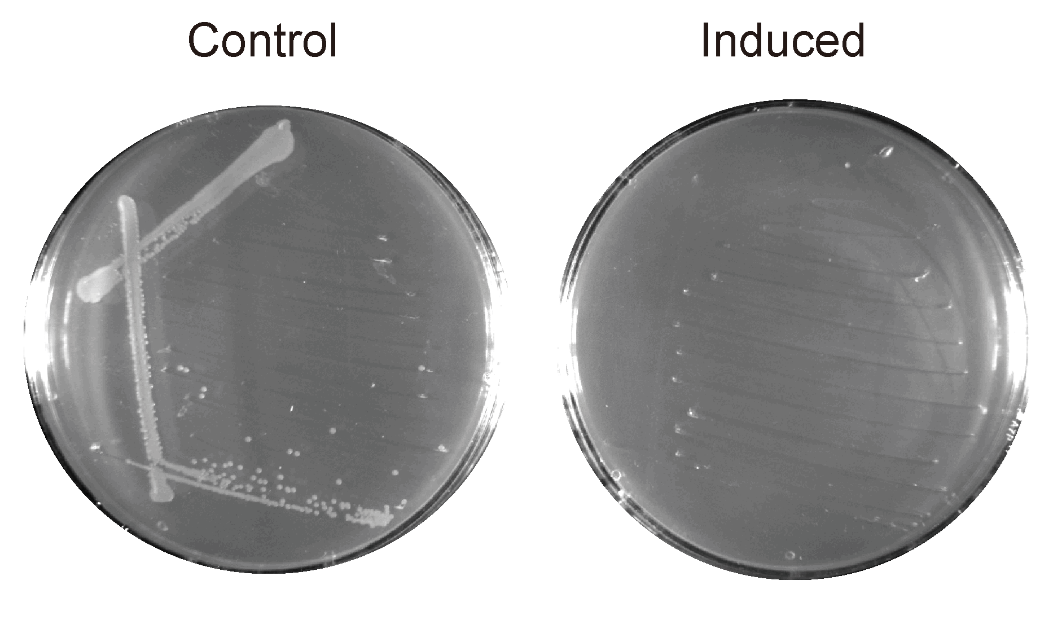

Supplement: S5 Fig — Shown is growth of Δtdh ΔvqmAVc V. cholerae harboring phage VP882 vqmAPhage::Tn5 as a lysogen and arabinose-inducible 3xFLAG-VqmAPhage streaked onto agar plates with no arabinose (Control) or 0.2% arabinose (Induced). (TIF) [file pgen.1009550.s005.tif]

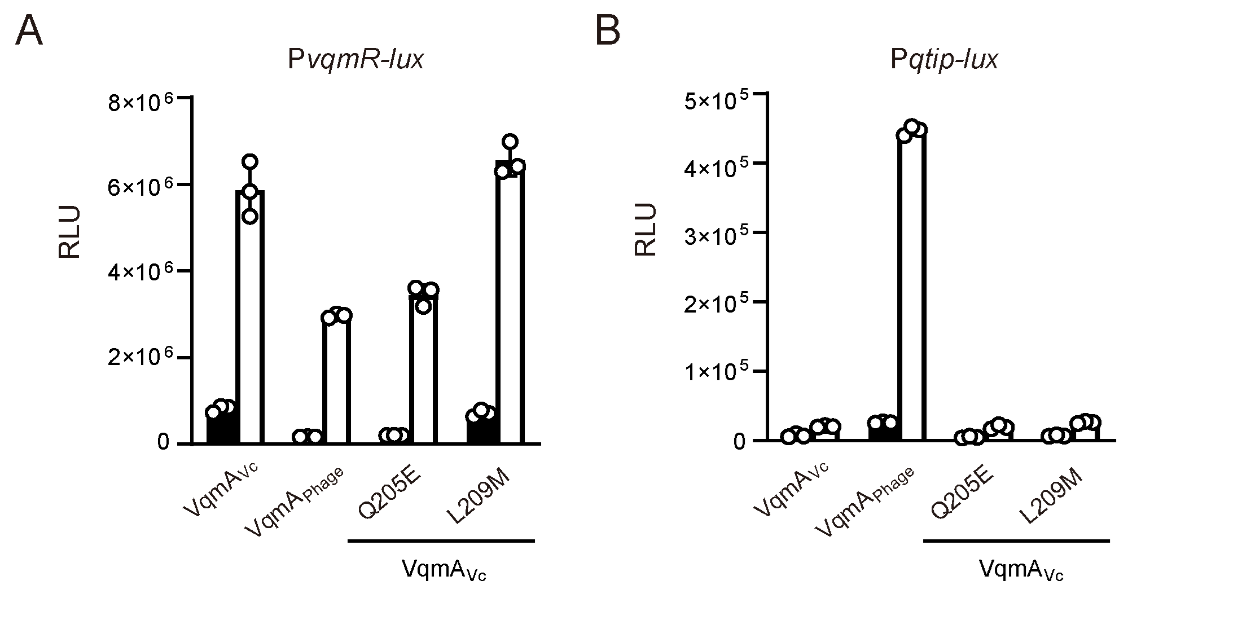

Supplement: S6 Fig — (A and B) Normalized reporter activity from Δtdh E. coli harboring (A) PvqmR-lux or (B) Pqtip-lux and arabinose-inducible 3xFLAG-VqmAVc, 3xFLAG-VqmAPhage, or the indicated 3xFLAG-VqmAVc allele. Black, no arabinose; white, 0.2% arabinose. Data are represented as mean ± SD (error bars) with n = 3 biological replicates. (TIF) [file pgen.1009550.s006.tif]

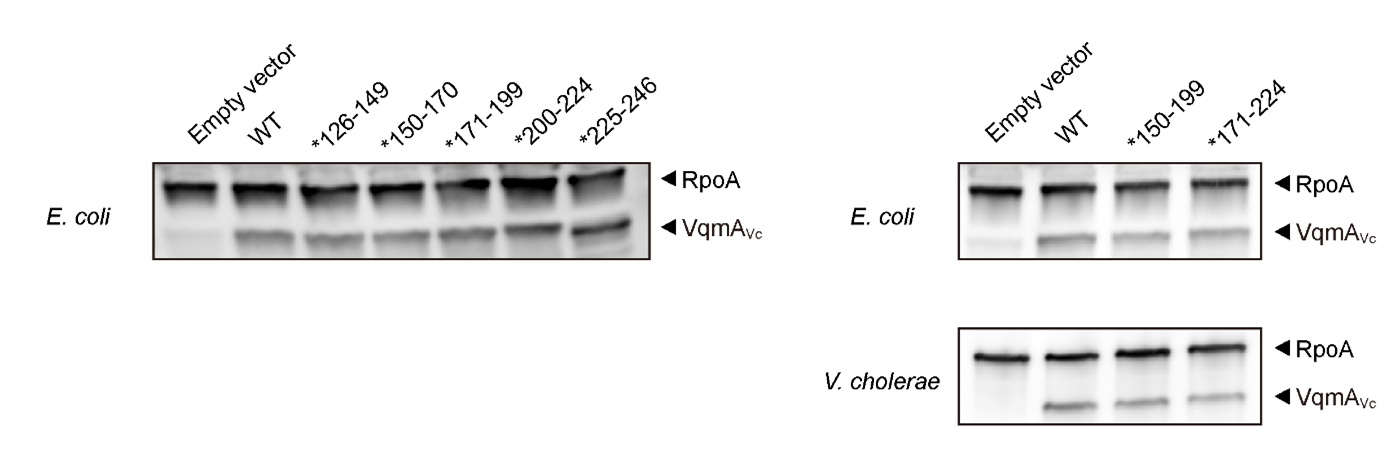

Supplement: S7 Fig — Western blot showing the designated 3xFLAG-VqmAVc mosaic proteins produced by Δtdh E. coli and Δtdh ΔvqmAVc V. cholerae. RpoA was used as the loading control. Data are representative of two independent experiments. (TIF) [file pgen.1009550.s007.tif]

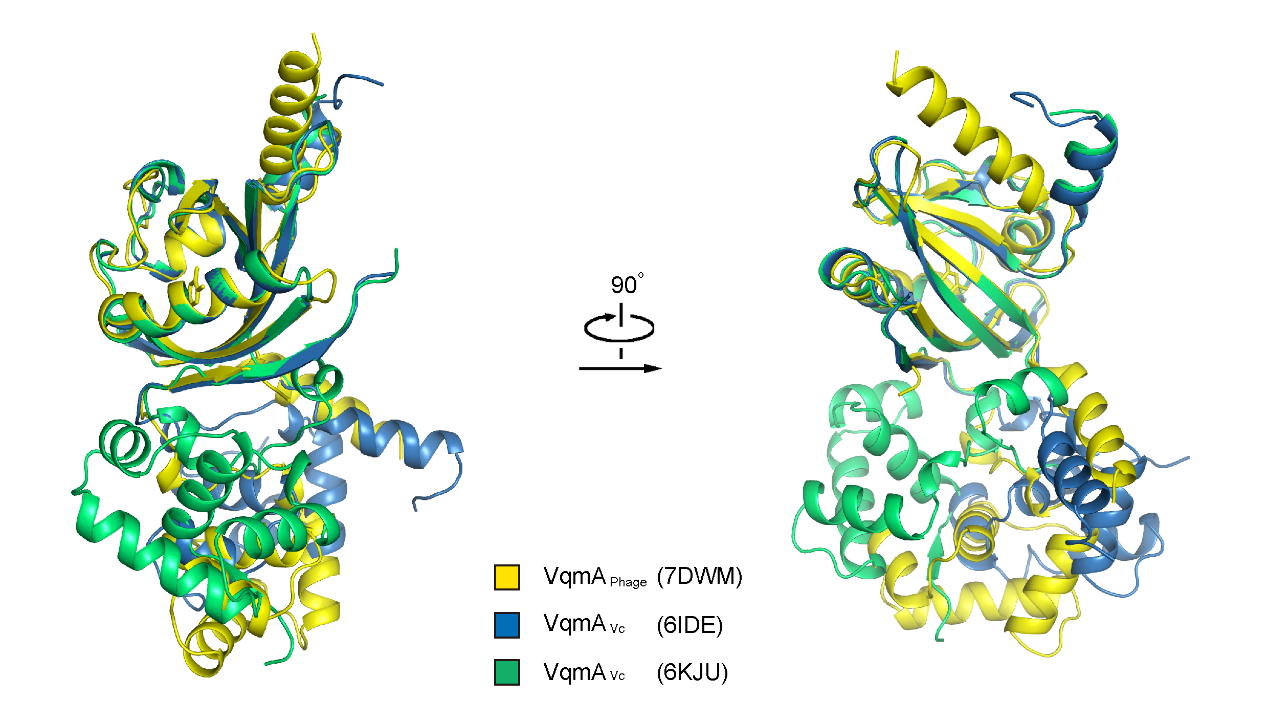

Supplement: S8 Fig — Previously reported crystal structures of DPO-VqmAVc-PvqmR (blue, PDB: 6IDE) and DPO-VqmAVc (green, PDB: 6KJU) superimposed onto the recently published crystal structure of DPO-VqmAPhage (yellow, PDB: 7DWM) based on the orientations of the PAS domains. DNA in the DPO-VqmAVc-PvqmR structure was omitted for simplicity. (TIF) [file pgen.1009550.s008.tif]

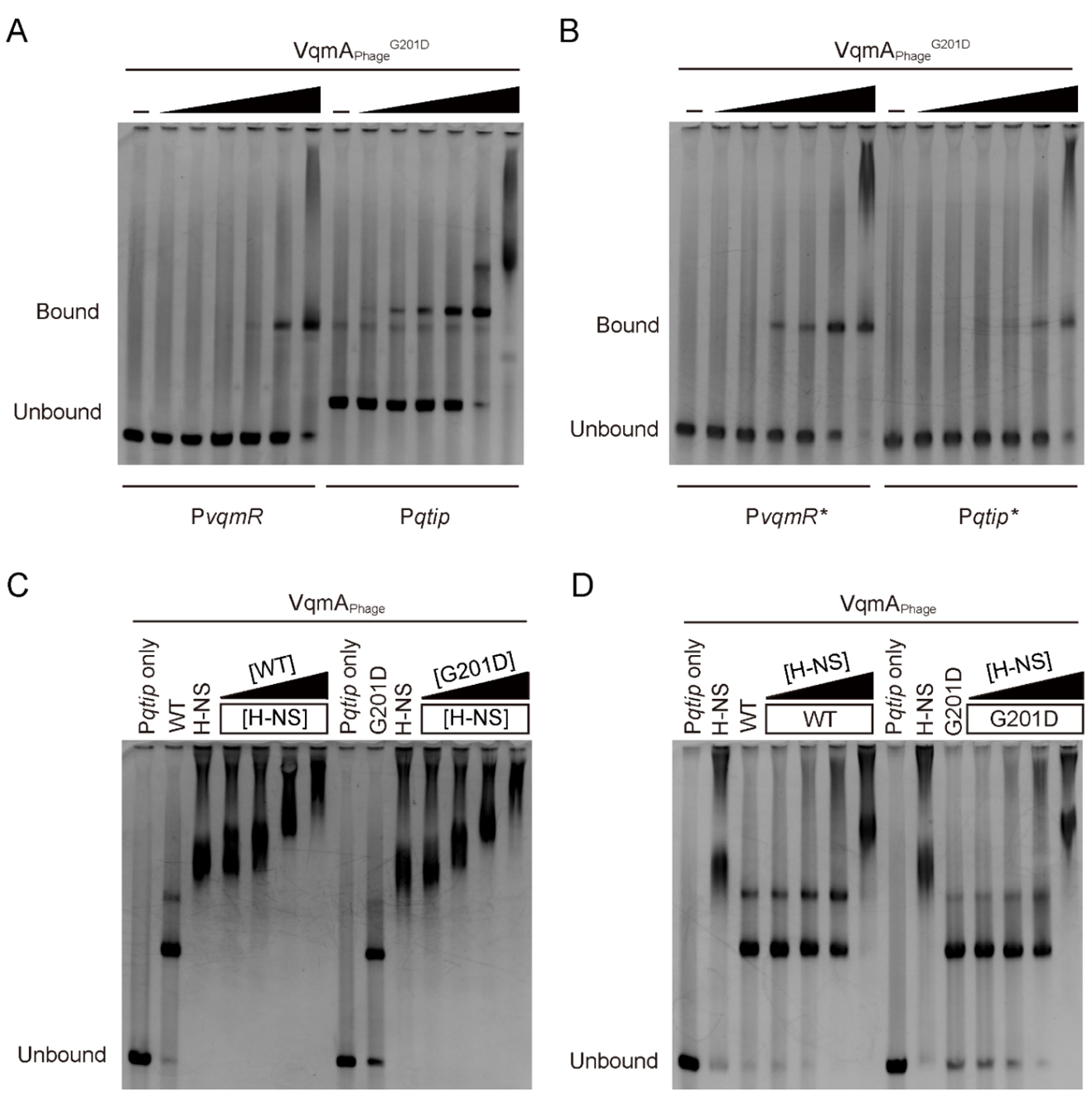

Supplement: S9 Fig — (A) EMSA showing binding of VqmAPhageG201D to PvqmR and Pqtip DNA. 25 nM DNA was used in all EMSAs with no protein (designated -) or 2-fold serial dilutions of proteins. The lowest and highest protein (dimer) concentrations are 18.75 nM and 600 nM, respectively. (B) As in panel A for PvqmR* and Pqtip* DNA. (C) EMSA showing WT VqmAPhage and VqmAPhageG201D binding to Pqtip DNA in the presence of H-NS (300 nM). (D) EMSA showing H-NS binding to Pqtip DNA in the presence of WT VqmAPhage or VqmAPhageG201D (each protein at 300 nM). (TIF) [file pgen.1009550.s009.tif]

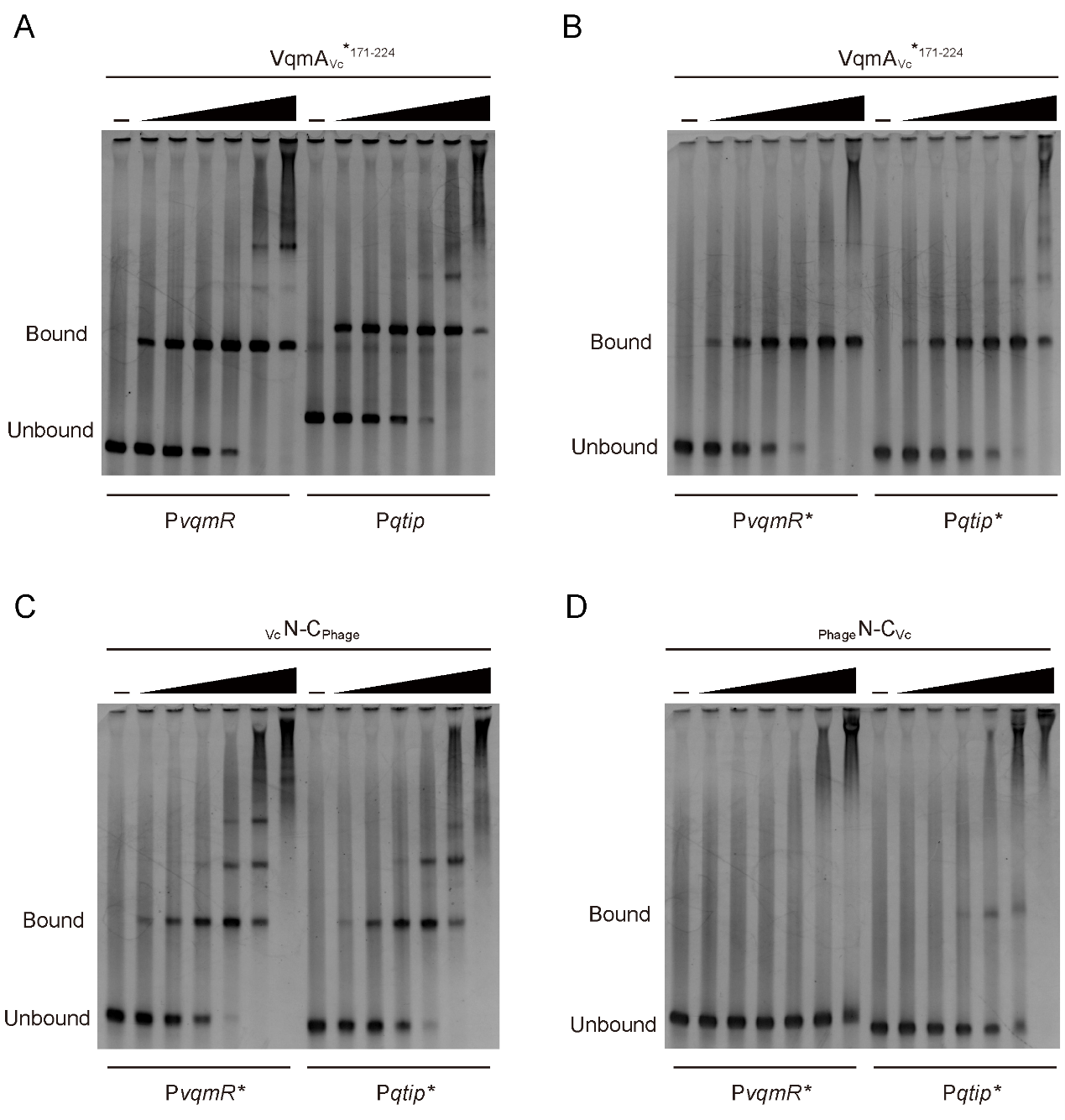

Supplement: S10 Fig — (A) EMSA showing binding of VqmAVc*171–224 to PvqmR and Pqtip DNA. 25 nM DNA was used in all EMSAs with no protein (designated -) or 2-fold serial dilutions of proteins. The lowest and highest protein (dimer) concentrations are 18.75 nM and 600 nM, respectively. (B) As in panel A for PvqmR* and Pqtip* DNA. (C) As in panel A for VcN-CPhage binding to PvqmR* and Pqtip* DNA. (D) As in panel C for PhageN-CVc. (TIF) [file pgen.1009550.s010.tif]
