# Supplementary material for: Mechanism underlying the DNA-binding preferences of the Vibrio cholerae and vibriophage VP882 VqmA quorum-sensing receptors
Source: PLoS Genet. 2021 Jul 6;17(7):e1009550. doi: 10.1371/journal.pgen.1009550 (PMC8284805; doi:10.1371/journal.pgen.1009550)
Supplement: S1 Table — (DOCX) [file pgen.1009550.s011.docx]

**S1 Table. Bacterial strains used in this study**

| Strain | Genotype | | Purpose | Source |
| --- | --- | --- | --- | --- |
| ***V. cholerae* str. C6706** |  |  |  |  |
| JSS-852 | Δ*tdh* Δ*vqmA_Vc_,* P*vqmR*-*lux*::*lacZ* | | Western blot | [1] |
| JSS-1262 | Δ*tdh* Δ*vqmA_Vc_,* P*vqmR*-*lux*::*lacZ*; VP882 (*vqmA_Phage_*::Tn*5*) | | Growth and lysis assays | This study |
| ***E. coli* BW25113** |  | |  |  |
| VcOD-35 | *lacIq*, *rrnBT14*, Δ*lacZWJ16*, *hsdR514*, Δ*araBADAH33*, Δ*rhaBADLD78*, Δ*tdh* | | Bioluminescence assays, Western blot | [1,2] |
| ***E. coli* TOP10** | F- *mcrA* Δ(*mrr*-*hsdRMS*-*mcrBC*) Φ80*lacZ*Δ*M15* Δ*lacX74 recA1 araD139* Δ(*ara leu*)*7697* *galU galK rpsL* (Str^R^) *endA1 nupG* | | Cloning, bioluminescence assays | Invitrogen |
| ***E. coli* BL21 (DE3)** |  | |  |  |
| JSS-915 | str. B, F- *ompT hsdSB (rBmB*-*) gal dcm (DE3)*, *tdh*::*kan^R^* | | Protein expression and purification | Invitrogen, [1] |

**S1 Table References**

1. Huang X, Duddy OP, Silpe JE, Paczkowski JE, Cong J, Henke BR, *et al*. Mechanism underlying autoinducer recognition in the *Vibrio cholerae* DPO-VqmA quorum-sensing pathway. *J Biol Chem*. 2020 Mar 6;295(10):2916–31.
2. Baba T, Ara T, Hasegawa M, Takai Y, Okumura Y, Baba M, *et al.* Construction of *Escherichia coli* K-12 in-frame, single-gene knockout mutants: The Keio collection. *Mol Syst Biol.* 2006 May 16;2.
