# Supplementary material for: Mechanism underlying the DNA-binding preferences of the Vibrio cholerae and vibriophage VP882 VqmA quorum-sensing receptors
Source: PLoS Genet. 2021 Jul 6;17(7):e1009550. doi: 10.1371/journal.pgen.1009550 (PMC8284805; doi:10.1371/journal.pgen.1009550)
Supplement: S2 Table — (DOCX) [file pgen.1009550.s012.docx]

**S2 Table. Plasmids used in this study**

|  | |  |  |  |
| --- | --- | --- | --- | --- |
| Plasmid Name | Plasmid ID | Marker, Origin | Ribosome Binding Site | Source |
| **Bioluminescence, Growth, and Lysis Assays** |  |  |  |  |
| pBR322-P*vqmR*-*lux* | pJES-101 | Amp, pBR322 | N/A | [1] |
| pBR322-P*qtip*-*lux* | pJES-119 | Amp, pBR322 | native *qtip* | [1] |
| pBR322-P*vqmR**-*lux* | pXH-1 | Amp, pBR322 | N/A | This study |
| pBR322-P*qtip**-*lux* | pXH-2 | Amp, pBR322 | native *qtip* | This study |
| pEVS-pBAD empty | pJY-014 | Kan, p15A | N/A | [2] |
| pEVS-pBAD-*vqmA_Vc_* | pKP-375 | Kan, p15A | native *vqmA_Vc_* | [3] |
| pEVS-pBAD-*vqmA_Phage_* | pOD-7 | Kan, p15A | native *vqmA_Vc_* | This study |
| pEVS-pBAD-*_Vc_N*-*C_Phage_* | pOD-8 | Kan, p15A | native *vqmA_Vc_* | This study |
| pEVS-pBAD-*_Phage_N*-*C_Vc_* | pOD-9 | Kan, p15A | native *vqmA_Vc_* | This study |
| pEVS-pBAD-*3xFLAG*-*vqmA_Vc_* | pOD-10 | Kan, p15A | native *vqmA_Vc_* | This study |
| pEVS-pBAD-*3xFLAG*-*vqmA_Phage_* | pOD-11 | Kan, p15A | native *vqmA_Vc_* | This study |
| pEVS-pBAD-*DBD_Vc_* | pXH-3 | Kan, p15A | native *vqmA_Vc_* | This study |
| pEVS-pBAD-*DBD_Phage_* | pXH-4 | Kan, p15A | native *vqmA_Vc_* | This study |
| pEVS-pBAD-*GST*-*DBD_Vc_* | pXH-5 | Kan, p15A | native *vqmA_Vc_* | This study |
| pEVS-pBAD-*GST*-*DBD_Phage_* | pXH-6 | Kan, p15A | native *vqmA_Vc_* | This study |
| pEVS-pBAD-*3xFLAG*-*vqmA_Phage_ K176Q* | pOD-12 | Kan, p15A | native *vqmA_Vc_* | This study |
| pEVS-pBAD-*3xFLAG*-*vqmA_Phage_ R184I* | pOD-13 | Kan, p15A | native *vqmA_Vc_* | This study |
| pEVS-pBAD-*3xFLAG*-*vqmA_Phage_ I193E* | pOD-14 | Kan, p15A | native *vqmA_Vc_* | This study |
| pEVS-pBAD-*3xFLAG*-*vqmA_Phage_ E194A* | pOD-15 | Kan, p15A | native *vqmA_Vc_* | This study |
| pEVS-pBAD-*3xFLAG*-*vqmA_Phage_ K176Q, R184I, I193E, E194A* | pOD-16 | Kan, p15A | native *vqmA_Vc_* | This study |
| pEVS-pBAD-*3xFLAG*-*vqmA_Phage_ G201D* | pOD-17 | Kan, p15A | native *vqmA_Vc_* | This study |
| pEVS-pBAD-*3xFLAG*-*vqmA_Phage_ G201R* | pOD-18 | Kan, p15A | native *vqmA_Vc_* | This study |
| pEVS-pBAD-*3xFLAG*-*vqmA_Phage_ A202V* | pOD-19 | Kan, p15A | native *vqmA_Vc_* | This study |
| pEVS-pBAD-*3xFLAG*-*vqmA_Phage_ E207K* | pOD-20 | Kan, p15A | native *vqmA_Vc_* | This study |
| pEVS-pBAD-*3xFLAG*-*vqmA_Phage_ E207V* | pOD-21 | Kan, p15A | native *vqmA_Vc_* | This study |
| pEVS-pBAD-*3xFLAG*-*vqmA_Phage_ M211K* | pOD-22 | Kan, p15A | native *vqmA_Vc_* | This study |
| pEVS-pBAD-*3xFLAG*-*vqmA_Vc_ Q174K* | pOD-23 | Kan, p15A | native *vqmA_Vc_* | This study |
| pEVS-pBAD-*3xFLAG*-*vqmA_Vc_ I182R* | pOD-24 | Kan, p15A | native *vqmA_Vc_* | This study |
| pEVS-pBAD-*3xFLAG*-*vqmA_Vc_ E191I* | pOD-25 | Kan, p15A | native *vqmA_Vc_* | This study |
| pEVS-pBAD-*3xFLAG*-*vqmA_Vc_ A192E* | pOD-26 | Kan, p15A | native *vqmA_Vc_* | This study |
| pEVS-pBAD-*3xFLAG*-*vqmA_Vc_ Q174K, I182R, E191I, A192E* | pOD-27 | Kan, p15A | native *vqmA_Vc_* | This study |
| pEVS-pBAD-*3xFLAG*-*vqmA_Vc_ Q205E* | pOD-28 | Kan, p15A | native *vqmA_Vc_* | This study |
| pEVS-pBAD-*3xFLAG*-*vqmA_Vc_ L209M* | pOD-29 | Kan, p15A | native *vqmA_Vc_* | This study |
| pEVS-pBAD-*vqmA_Vc_* **126*-*149* | pOD-30 | Kan, p15A | native *vqmA_Vc_* | This study |
| pEVS-pBAD-*vqmA_Vc_* **150*-*170* | pOD-31 | Kan, p15A | native *vqmA_Vc_* | This study |
| pEVS-pBAD-*vqmA_Vc_* **171*-*199* | pOD-32 | Kan, p15A | native *vqmA_Vc_* | This study |
| pEVS-pBAD-*vqmA_Vc_* **220*-*224* | pOD-33 | Kan, p15A | native *vqmA_Vc_* | This study |
| pEVS-pBAD-*vqmA_Vc_* **225*-*246* | pOD-34 | Kan, p15A | native *vqmA_Vc_* | This study |
| pEVS-pBAD-*vqmA_Vc_* **150*-*199* | pOD-35 | Kan, p15A | native *vqmA_Vc_* | This study |
| pEVS-pBAD-*vqmA_Vc_* **171*-*224* | pOD-36 | Kan, p15A | native *vqmA_Vc_* | This study |
| pEVS-pBAD-*3xFLAG*-*vqmA_Vc_* **126*-*149* | pOD-37 | Kan, p15A | native *vqmA_Vc_* | This study |
| pEVS-pBAD-*3xFLAG*-*vqmA_Vc_* **150*-*170* | pOD-38 | Kan, p15A | native *vqmA_Vc_* | This study |
| pEVS-pBAD-*3xFLAG*-*vqmA_Vc_* **171*-*199* | pOD-39 | Kan, p15A | native *vqmA_Vc_* | This study |
| pEVS-pBAD-*3xFLAG*-*vqmA_Vc_* **220*-*224* | pOD-40 | Kan, p15A | native *vqmA_Vc_* | This study |
| pEVS-pBAD-*3xFLAG*-*vqmA_Vc_* **225*-*246* | pOD-41 | Kan, p15A | native *vqmA_Vc_* | This study |
| pEVS-pBAD-*3xFLAG*-*vqmA_Vc_* **150*-*199* | pOD-42 | Kan, p15A | native *vqmA_Vc_* | This study |
| pEVS-pBAD-*3xFLAG*-*vqmA_Vc_* **171*-*224* | pOD-43 | Kan, p15A | native *vqmA_Vc_* | This study |
| **Protein expression and purification** |  |  |  |  |
| pET15b-pT7-*vqmA_Vc_* | pJES-75 | Amp, pBR322 | T7 leader | [4] |
| pET15b-pT7-*vqmA_Phage_* | pXH-7 | Amp, pBR322 | T7 leader | This study |
| pET15b-pT7-*_Vc_N*-*C_Phage_* | pXH-8 | Amp, pBR322 | T7 leader | This study |
| pET15b-pT7-*_Phage_N*-*C_Vc_* | pXH-9 | Amp, pBR322 | T7 leader | This study |
| pET15b-pT7-*DBD_Vc_* | pXH-10 | Amp, pBR322 | T7 leader | This study |
| pET15b-pT7-*DBD_Phage_* | pXH-11 | Amp, pBR322 | T7 leader | This study |
| pGEX-pT7-*GST*-*DBD_Vc_* | pXH-12 | Amp, pBR322 | T7 leader | This study |
| pGEX-pT7-*GST-DBD_Phage_* | pXH-13 | Amp, pBR322 | T7 leader | This study |

**S2 Table References**

1. Silpe JE, Bassler BL. A host-produced quorum-sensing autoinducer controls a phage lysis-lysogeny decision. *Cell*. 2019 Jan 10;176(1–2):268-280.e13.
2. Yan J, Sharo AG, Stone HA, Wingreen NS, Bassler BL. *Vibrio cholerae* biofilm growth program and architecture revealed by single-cell live imaging. *Proc Natl Acad Sci U S A*. 2016 Sep 6;113(36):e5337–43.
3. Papenfort K, Förstner KU, Cong JP, Sharma CM, Bassler BL. Differential RNA-seq of *Vibrio cholerae* identifies the VqmR small RNA as a regulator of biofilm formation. *Proc Natl Acad Sci U S A*. 2015 Feb 17;112(7):E766–75.
4. Huang X, Duddy OP, Silpe JE, Paczkowski JE, Cong J, Henke BR, *et al*. Mechanism underlying autoinducer recognition in the *Vibrio cholerae* DPO-VqmA quorum-sensing pathway. *J Biol Chem*. 2020 Mar 6;295(10):2916–31.
