# Supplementary material for: Mechanism underlying the DNA-binding preferences of the Vibrio cholerae and vibriophage VP882 VqmA quorum-sensing receptors
Source: PLoS Genet. 2021 Jul 6;17(7):e1009550. doi: 10.1371/journal.pgen.1009550 (PMC8284805; doi:10.1371/journal.pgen.1009550)
Supplement: S3 Table — (DOCX) [file pgen.1009550.s013.docx]

**S3 Table. Primers used in this study**

| Primer | Sequence (5' - 3') | *5' | Purpose |
| --- | --- | --- | --- |
| **Plasmid Construction** |  |  |  |
| ODO-115 | GCCATATCCTCCACTGGAAATGCG |  | pEVS-pBAD backbone antisense |
| ODO-523 | TTTATCGTCATCTTTGTAGTCGATATCATG |  | pEVS-pBAD-*3xFLAG* backbone antisense |
| ODO-2 | TAAGCAACAACGTCAAGCTGATTG |  | pEVS-pBAD backbone sense |
| ODO-511 | CGCAGTGTCGGTTAAATCTTGC |  | *vqmA_Vc_* internal antisense |
| XH-1 | CATGCCATGGGCATGCCTAACCATCTG |  | *vqmA_Vc_*, pET15b insert sense |
| XH-2 | CATGCCATGGGCATGACATTTAAATCGGTCG |  | *DBD_Vc_*, pET15b and pGEX insert sense |
| XH-3 | CTACACTCGAGTTACTGGGCGACAACGTC |  | *vqmA_Vc_* and *DBD_Vc_*, pET15b and pGEX insert antisense |
| XH-4 | CATGCCATGGGCATGTCAATAAGCGAAGG |  | *vqmA_Phage_*, pET15b insert sense |
| XH-5 | CATGCCATGGGCATGGGATCCTTCGAGACC |  | *DBD_Phage_*, pET15b and pGEX insert sense |
| XH-6 | CTACACTCGAGTTACTTGAGCAGCATCGAG |  | *vqmA_Phage_* and *DBD_Phage_*, pET15b and pGEX insert antisense |
| ODO-371 | GCCGAACGGCCCAGGATATCGCTGG | 5'P | *vqmA_Phage_ K176Q* site-directed mutagenesis sense |
| ODO-372 | CACGAAGCAGGAAGAACAGCACCAGT |  | *vqmA_Phage_ K176Q* site-directed mutagenesis antisense |
| ODO-373 | ATGCTGGGGATCTCTCCCCGCACCA | 5'P | *vqmA_Phage_ R184I* site-directed mutagenesis sense |
| ODO-374 | GCCAGCGATATCCTTGGCCGTTCGG |  | *vqmA_Phage_ R184I* site-directed mutagenesis antisense |
| ODO-375 | AACACGCTGAAGAGCGCATCCGCAA | 5'P | *vqmA_Phage_ I193E* site-directed mutagenesis sense |
| ODO-377 | AACACGCTATCGCGCGCATCCGCAA | 5'P | *vqmA_Phage_ E194A* site-directed mutagenesis sense |
| ODO-376 | CGATGGTGCGGGGAGAGCGCCCCAG |  | *vqmA_Phage_ I193E/E194A* site-directed mutagenesis antisense |
| ODO-388 | ACAAATTCGGTGTTGGCAACAAGC | 5'P | *vqmA_Phage_ A202V* site-directed mutagenesis sense |
| ODO-389 | TGCGGATGCGCTCGATAGCGTGTT |  | *vqmA_Phage_ A202V* site-directed mutagenesis antisense |
| ODO-418 | ACAAATTCGATGCTGGCAACAAGCG | 5'P | *vqmA_Phage_ G201D* site-directed mutagenesis sense |
| ODO-401 | ACAAATTCCGTGCTGGCAACAAGCG | 5'P | *vqmA_Phage_ G201R* site-directed mutagenesis sense |
| ODO-402 | TGCGGATGCGCTCGATAGCGTGTTC |  | *vqmA_Phage_ G201D/R* site-directed mutagenesis antisense |
| ODO-407 | GCAACAAGCGGGTGCTCATCGATATGG | 5'P | *vqmA_Phage_ E207V* site-directed mutagenesis sense |
| ODO-408 | CAGCACCGAATTTGTTGCGGATGCGC |  | *vqmA_Phage_ E207K/V* site-directed mutagenesis antisense |
| ODO-409 | GCAACAAGCGGAAGCTCATCGATATGG | 5'P | *vqmA_Phage_ E207K* site-directed mutagenesis sense |
| ODO-428 | GGAGCTCATCGATAAGGCCATGTCCAA | 5'P | *vqmA_Phage_ M211K* site-directed mutagenesis sense |
| ODO-381 | CGCTTGTTGCCAGCACCGAATTTGT |  | *vqmA_Phage_ M211K* site-directed mutagenesis antisense |
| XH-15 | AAAAAACCGAAGCACATTGCACG | 5'P | *vqmA_Vc_ Q174K* site-directed mutagenesis sense |
| XH-16 | ACCGTATAAGAGCAGAAATAG |  | *vqmA_Vc_ Q174K* site-directed mutagenesis antisense |
| XH-17 | AGTAATGGGGCGTTCGATTAAAAC | 5'P | *vqmA_Vc_ I182R* site-directed mutagenesis sense |
| XH-18 | CGTGCAATGTGCTGCGGTT |  | *vqmA_Vc_ I182R* site-directed mutagenesis antisense |
| ODO-520 | AAGGGTATATTGCCAAACTACGCA | 5'P | *vqmA_Vc_ E191I* site-directed mutagenesis sense |
| ODO-522 | CCACCGTTTTAATCGAAATCCCCATTACT |  | *vqmA_Vc_ E191I/A192E* site-directed mutagenesis antisense |
| ODO-521 | AAGGGTATGAGGAAAAACTACGCA | 5'P | *vqmA_Vc_ A192E* site-directed mutagenesis sense |
| ODO-516 | AGCAAAGACGAACTGATTGACCTTG | 5'P | *vqmA_Vc_ Q205E* site-directed mutagenesis sense |
| ODO-517 | CAATGCCCCAAATTTACTGCGTAGT |  | *vqmA_Vc_ Q205E* site-directed mutagenesis antisense |
| ODO-518 | ACCAACTGATTGACATGGCTTTAGATC | 5'P | *vqmA_Vc_ L209M* site-directed mutagenesis sense |
| ODO-519 | CTTTGCTCAATGCCCCAAATTTACTG |  | *vqmA_Vc_ L209M* site-directed mutagenesis antisense |
| XH-7 | CCAATTATGTCGGTTTCCGAGTATTGCGCG |  | P*vqmR**, pBR322 insert sense |
| XH-8 | CGGCCGCATGCTCTGGTTTGTACTTTACCG |  | P*vqmR**, pBR322 insert antisense |
| XH-9 | CGGTAAAGTACAAACCAGAGCATGCGGCCG |  | pBR322-P*vqmR*-*lux* backbone sense |
| XH-10 | CGCGCAATACTCGGAAACCGACATAATTGG |  | pBR322-P*vqmR*-*lux* backbone antisense |
| XH-11 | ATACACTCCTTGTAAGTGATTGTTATAAGG |  | P*qtip**, pBR322 insert sense |
| XH-12 | ATCATCCCCTTCGCTTATTGACATTTATCA |  | P*qtip**, pBR322 insert antisense |
| XH-13 | TGATAAATGTCAATAAGCGAAGGGGATGAT |  | pBR322-P*qtip*-*lux* backbone sense |
| XH-14 | CCTTATAACAATCACTTACAAGGAGTGTAT |  | pBR322-P*qtip*-*lux* backbone antisense |
| XH-19 | CAAAGATGACGATAAATCCCCTATACTAGGT |  | *GST*-*DBD_Vc_* and *GST*-*DBD_Phage_*, pEVS-pBAD insert sense |
| XH-20 | CAAAGATGACGATAAAACATTTAAATCGGTC |  | *DBD_Vc_*, pEVS-pBAD insert sense |
| XH-21 | CAAAGATGACGATAAAGGATCCTTCGAGACC |  | *DBD_Phage_*, pEVS-pBAD insert sense |
| XH-22 | CGGATCCTGGTTGCGCTACTGGGCGACAAC |  | *DBD_Vc_* and *GST*-*DBD_Vc_*, pEVS-pBAD insert antisense |
| XH-23 | CGGATCCTGGTTGCGCTACTTGAGCAGCAT |  | *DBD_Phage_* and *GST*-*DBD_Phage_*, pEVS-pBAD insert antisense |
| XH-24 | GTTGTCGCCCAGTAGCGCAACCAGGATCCG |  | pEVS-pBAD backbone, *DBD_Vc_* and *GST*-*DBD_Vc_* homology sense |
| XH-25 | ATGCTGCTCAAGTAGCGCAACCAGGATCCG |  | pEVS-pBAD backbone, *DBD_Phage_* and *GST*-*DBD_Phage_* homology sense |
| XH-26 | ACCTAGTATAGGGGATTTATCGTCATCTTTG |  | pEVS-pBAD backbone, *GST*-*DBD_Vc_* and *GST*-*DBD_Phage_* homology antisense |
| XH-27 | GACCGATTTAAATGTTTTATCGTCATCTTTG |  | pEVS-pBAD backbone, *DBD_Phage_* homology antisense |
| XH-28 | GGTCTCGAAGGATCCTTTATCGTCATCTTTG |  | pEVS-pBAD backbone, *DBD_Vc_* homology antisense |
| **Random Mutagenesis** |  |  |  |
| ODO-432 | TTCTTCGGGCAAGATTTAACC |  | *vqmA_Vc_* internal sense |
| ODO-433 | TCTAAGCAAGGTTTTCGGGAT |  | *vqmA_Vc_* internal antisense |
| JSO-1680 | TTCCACGGACAAGACCTGACT |  | *vqmA_Phage_* internal sense |
| JSO-1682 | GTGAAACAGGGCTTTTGGCAC |  | *vqmA_Phage_* internal antisense |
| **EMSA** |  |  |  |
| VR100 | TCCGAGTATTGCGCGAGCTG |  | P*vqmR* sense |
| VR100-Rev | GTTTGTACTTTACCGAACGC |  | P*vqmR* antisense |
| JSO-1089 | GCATCATCCCCTTCGCTTATTGAC |  | P*qtip* sense |
| JSO-0897 | GAATACACTCCTTGTAAGTGATTGTTATAAGGAGC |  | P*qtip* antisense |
| PvqmR-Phage18-F* | TCCGAGTATTGCGCGAGCTGTAATGTTGACTCAAACAATTATGCATAAAGGGGGGAAAAATACCCTTTTTCATTTGTACCGCGTTCGGTAAAGTACAAAC |  | P*vqmR** sense (annealing) |
| PvqmR-Phage18-R* | GTTTGTACTTTACCGAACGCGGTACAAATGAAAAAGGGTATTTTTCCCCCCTTTATGCATAATTGTTTGAGTCAACATTACAGCTCGCGCAATACTCGGA |  | P*vqmR** antisense (annealing) |
| Pqtip-VR18-F* | GTGATTGTTATAAGGAGCTTTATTCTTGATGCATATAAGAAATACCGATAGGGGGATTTCCCCCCTGATAAGCAGCTGCTAAAATTGATAAATGTCAATA |  | P*qtip** sense (annealing) |
| Pqtip-VR18-R* | TATTGACATTTATCAATTTTAGCAGCTGCTTATCAGGGGGGAAATCCCCCTATCGGTATTTCTTATATGCATCAAGAATAAAGCTCCTTATAACAATCAC |  | P*qtip** antisense (annealing) |

*5’ modification designation: 5’P; Phosphorylation
