# Supplementary material for: Mechanism underlying the DNA-binding preferences of the Vibrio cholerae and vibriophage VP882 VqmA quorum-sensing receptors
Source: PLoS Genet. 2021 Jul 6;17(7):e1009550. doi: 10.1371/journal.pgen.1009550 (PMC8284805; doi:10.1371/journal.pgen.1009550)
Supplement: S4 Table — (DOCX) [file pgen.1009550.s014.docx]

**S4 Table. gBlocks used in this study**

| gBlock name | Sequence (5' - 3') | Purpose |
| --- | --- | --- |
| **Plasmid Construction** |  |  |
| JSgBlock-123b | CTCTCTACTGTTTCTCCGCATTTCCAGTGGAGGATATGGCGTGCCTAACCATCTGACATTAGAGCAGATTTCTTTATTCAAACAATTACCCGGTTATTGGGGATGCAAGGACTTAAACTCGGTTTTTGTGTATGCCAACCAAGCCTATGGCGAATTGATCGGCTTAAAGCGCGCTGAAGATTGCATCGGGCGCACCGATTTTGAAATGCCTAGCCCAACAGCAGCTTGTGCTGCCGAATTTCAACAGCAAGATCGCTATGTGATTGAAACGGGGCATTCCGTCAAAGTGCTCGATATTCATCCTTACCCTGATGGCCACTGGCATGCGCATATTTTTACCAAGACACCGTGGCGTGATAGCCAAGGCAAGATCCAAGGCACCATTTTCTTCGGGCAAGATTTAACCGACACTGCGGGCCGCATCGAGCGTGCAGTGGTTGAGCTGCTGCTGCCTTCCAGTGGCCAGGCTGGATCCTTCGAGACCAATGTGGTCGGTCTCAACTTGACCGAACGCGAGGAACTGGTGCTGTTCTTCCTGCTTCGTGGCCGAACGGCCAAGGATATCGCTGGCATGCTGGGGCGCTCTCCCCGCACCATCGAACACGCTATCGAGCGCATCCGCAACAAATTCGGTGCTGGCAACAAGCGGGAGCTCATCGATATGGCCATGTCCAAGGGTTATTACAGCATGGTGCCAAAAGCCCTGTTTCACACACAGGTCTCGATGCTGCTC | *_Vc_N*-*C_Phage_* construction |
| JSgBlock-124b | CTCTCTACTGTTTCTCCGCATTTCCAGTGGAGGATATGGCATGTCAATAAGCGAAGGGGATGATGCTTACATCCGCTCGTTGATTCATTTTTTTGGCAATCAACCGGATCCGTGGGGCATCAAGGACACCAAGTCGGTGTTCATCTATGCAAACCAGCCCTTTCGAGAGTTAGTCGGTATGAAGAACCGCAACGTGGAAGGACTTACCGACGCTGATATGGATTGCGAAACTGCGGCCTTTGCCGACTCCTTTCAGGCCCAAGATAGGCTGGTCGAGCAAGGCCGGGAGAAGAAAATCGTCCTGGACGTACACCCCTACGCGAATGGTTGGCGCGTTTTCACTTTCACCAAGACCCCTCTCATCATGCCGTCCGGACGTGTGGCCGGCACCATTTTCCACGGACAAGACCTGACTGACACGGCTATTCTTGAAGTTGGTCATTGGGTCTGCCGAGCAACTGGGTTATCCACCTCCACCACATTTAAATCGGTCGCCGATCGCGATACCTTGAAACTGACCGCGCGTGAATCAGAAGTCCTATTTCTGCTCTTATACGGTAAAAAACCGCAGCACATTGCACGAGTAATGGGGATTTCGATTAAAACGGTGGAAGGGTATGAGGCCAAACTACGCAGTAAATTTGGGGCATTGAGCAAAGACCAACTGATTGACCTTGCTTTAGATCGGGGATTCGGCTCAGTCATCCCGAAAACCTTGCTTAGAAAACAACTTTCCGTTGTGCTGAGCGATCACACGATCCCCAAAAAAGTTGACGTTGTCGCCCAG | *_Phage_N*-*C_Vc_* construction |
| ODgBlock-1 | GCATTTCCAGTGGAGGATATGGCATGGACTACAAAGACCATGACGGTGATTATAAAGATCATGATATCGACTACAAAGATGACGATAAACCTAACCATCTGACATTAGAGCAGATTTCTTTATTCAAACAATTACCCGGTTATTGGGGATGCAAGGACTTAAACTCGGTTTTTGTGTATGCCAACCAAGCCTATGGCGAATTGATCGGCTTAAAGCGCGCTGAAGATTGCATCGGGCGCACCGATTTTGAAATGCCTAGCCCAACAGCAGCTTGTGCTGCCGAATTTCAACAGCAAGATCGCTATGTGATTGAAACGGGGCATTCCGTCAAAGTGCTCGATATTCATCCTTACCCTGATGGCCACTGGCATGCGCATATTTTTACCAAGACACCGTGGCGTGATAGCCAAGGCAAGATCCAAGGCACCATTTTCTTCGGGCAAGATTTAACCGACACTGCGATTCTTGAAGTTGGTCATTGGGTCTGCCGAGCAACTGGGTTATCCACCTCCACCACATTTAAATCGGTCGCCGATCGCGATACCTTGAAACTGACCGCGCGTGAATCAGAAGTCCTATTTCTGCTCTTATACGGTAAAAAACCGCAGCACATTGCACGAGTAATGGGGATTTCGATTAAAACGGTGGAAGGGTATGAGGCCAAACTACGCAGTAAATTTGGGGCATTGAGCAAAGACCAACTGATTGACCTTGCTTTAGATCGGGGATTCGGCTCAGTCATCCCGAAAACCTTGCTTAGAAAACAACTTTCCGTTGTGCTGAGCGATCACACGATCCCCAAAAAAGTTGACGTTGTCGCCCAGTAAGCAACAACGTCAAGCTGATTG | *3xFLAG*-*vqmA_Vc_* construction |
| ODgBlock-2 | GCATTTCCAGTGGAGGATATGGCATGGACTACAAAGACCATGACGGTGATTATAAAGATCATGATATCGACTACAAAGATGACGATAAATCAATAAGCGAAGGGGATGATGCTTACATCCGCTCGTTGATTCATTTTTTTGGCAATCAACCGGATCCGTGGGGCATCAAGGACACCAAGTCGGTGTTCATCTATGCAAACCAGCCCTTTCGAGAGTTAGTCGGTATGAAGAACCGCAACGTGGAAGGACTTACCGACGCTGATATGGATTGCGAAACTGCGGCCTTTGCCGACTCCTTTCAGGCCCAAGATAGGCTGGTCGAGCAAGGCCGGGAGAAGAAAATCGTCCTGGACGTACACCCCTACGCGAATGGTTGGCGCGTTTTCACTTTCACCAAGACCCCTCTCATCATGCCGTCCGGACGTGTGGCCGGCACCATTTTCCACGGACAAGACCTGACTGACACGGCTGGCCGCATCGAGCGTGCAGTGGTTGAGCTGCTGCTGCCTTCCAGTGGCCAGGCTGGATCCTTCGAGACCAATGTGGTCGGTCTCAACTTGACCGAACGCGAGGAACTGGTGCTGTTCTTCCTGCTTCGTGGCCGAACGGCCAAGGATATCGCTGGCATGCTGGGGCGCTCTCCCCGCACCATCGAACACGCTATCGAGCGCATCCGCAACAAATTCGGTGCTGGCAACAAGCGGGAGCTCATCGATATGGCCATGTCCAAGGGTTATTACAGCATGGTGCCAAAAGCCCTGTTTCACACACAGGTCTCGATGCTGCTCAAGTAGTAAGCAACAACGTCAAGCTGATTG | *3xFLAG*-*vqmA_Phage_* construction |
| ODgBlock-3 | GCATTTCCAGTGGAGGATATGGCATGGACTACAAAGACCATGACGGTGATTATAAAGATCATGATATCGACTACAAAGATGACGATAAACCTAACCATCTGACATTAGAGCAGATTTCTTTATTCAAACAATTACCCGGTTATTGGGGATGCAAGGACTTAAACTCGGTTTTTGTGTATGCCAACCAAGCCTATGGCGAATTGATCGGCTTAAAGCGCGCTGAAGATTGCATCGGGCGCACCGATTTTGAAATGCCTAGCCCAACAGCAGCTTGTGCTGCCGAATTTCAACAGCAAGATCGCTATGTGATTGAAACGGGGCATTCCGTCAAAGTGCTCGATATTCATCCTTACCCTGATGGCCACTGGCATGCGCATATTTTTACCAAGACACCGTGGCGTGATAGCCAAGGCAAGATCCAAGGCACCATTTTCTTCGGGCAAGATTTAACCGACACTGCGATTCTTGAAGTTGGTCATTGGGTCTGCCGAGCAACTGGGTTATCCACCTCCACCACATTTAAATCGGTCGCCGATCGCGATACCTTGAAACTGACCGCGCGTGAATCAGAAGTCCTATTTCTGCTCTTATACGGTAAAAAACCGAAGCACATTGCACGAGTAATGGGGCGTTCGATTAAAACGGTGGAAGGGTATATTGAAAAACTACGCAGTAAATTTGGGGCATTGAGCAAAGACCAACTGATTGACCTTGCTTTAGATCGGGGATTCGGCTCAGTCATCCCGAAAACCTTGCTTAGAAAACAACTTTCCGTTGTGCTGAGCGATCACACGATCCCCAAAAAAGTTGACGTTGTCGCCCAGTAAGCAACAACGTCAAGCTGATTG | *3xFLAG*-*vqmA_Vc_ Q174K, I182R, E191I, A192E* construction |
| ODgBlock-4 | GCATTTCCAGTGGAGGATATGGCATGGACTACAAAGACCATGACGGTGATTATAAAGATCATGATATCGACTACAAAGATGACGATAAATCAATAAGCGAAGGGGATGATGCTTACATCCGCTCGTTGATTCATTTTTTTGGCAATCAACCGGATCCGTGGGGCATCAAGGACACCAAGTCGGTGTTCATCTATGCAAACCAGCCCTTTCGAGAGTTAGTCGGTATGAAGAACCGCAACGTGGAAGGACTTACCGACGCTGATATGGATTGCGAAACTGCGGCCTTTGCCGACTCCTTTCAGGCCCAAGATAGGCTGGTCGAGCAAGGCCGGGAGAAGAAAATCGTCCTGGACGTACACCCCTACGCGAATGGTTGGCGCGTTTTCACTTTCACCAAGACCCCTCTCATCATGCCGTCCGGACGTGTGGCCGGCACCATTTTCCACGGACAAGACCTGACTGACACGGCTGGCCGCATCGAGCGTGCAGTGGTTGAGCTGCTGCTGCCTTCCAGTGGCCAGGCTGGATCCTTCGAGACCAATGTGGTCGGTCTCAACTTGACCGAACGCGAGGAACTGGTGCTGTTCTTCCTGCTTCGTGGCCGAACGGCCCAGGATATCGCTGGCATGCTGGGGATCTCTCCCCGCACCATCGAACACGCTGAAGCGCGCATCCGCAACAAATTCGGTGCTGGCAACAAGCGGGAGCTCATCGATATGGCCATGTCCAAGGGTTATTACAGCATGGTGCCAAAAGCCCTGTTTCACACACAGGTCTCGATGCTGCTCAAGTAGTAAGCAACAACGTCAAGCTGATTG | *3xFLAG*-*vqmA_Phage_ K176Q, R184I, I193E, E194A* construction |
| ODgBlock-5 | GGCAAGATTTAACCGACACTGCGGGCCGCATCGAGCGTGCAGTGGTTGAGCTGCTGCTGCCTTCCAGTGGCCAGGCTGGATCCTTCGAGACCAATCGCGATACCTTGAAACTGACCGCGCGTGAATCAGAAGTCCTATTTCTGCTCTTATACGGTAAAAAACCGCAGCACATTGCACGAGTAATGGGGATTTCGATTAAAACGGTGGAAGGGTATGAGGCCAAACTACGCAGTAAATTTGGGGCATTGAGCAAAGACCAACTGATTGACCTTGCTTTAGATCGGGGATTCGGCTCAGTCATCCCGAAAACCTTGCTTAGAAAACAACTTTCCGTTGTGCTGAGCGATCACACGATCCCCAAAAAAGTTGACGTTGTCGCCCAGTAAGCAACAACGTCAAGCTGATTG | *vqmA_Vc_ *126*-*149* construction |
| ODgBlock-6 | GCAAGATTTAACCGACACTGCGATTCTTGAAGTTGGTCATTGGGTCTGCCGAGCAACTGGGTTATCCACCTCCACCACATTTAAATCGGTCGCCGATGTGGTCGGTCTCAACTTGACCGAACGCGAGGAACTGGTGCTGTTCTTCCTGCTTCGTGGCCGAAAACCGCAGCACATTGCACGAGTAATGGGGATTTCGATTAAAACGGTGGAAGGGTATGAGGCCAAACTACGCAGTAAATTTGGGGCATTGAGCAAAGACCAACTGATTGACCTTGCTTTAGATCGGGGATTCGGCTCAGTCATCCCGAAAACCTTGCTTAGAAAACAACTTTCCGTTGTGCTGAGCGATCACACGATCCCCAAAAAAGTTGACGTTGTCGCCCAGTAAGCAACAACGTCAAGCTGATTG | *vqmA_Vc_ *150*-*170* construction |
| ODgBlock-7 | GCAAGATTTAACCGACACTGCGATTCTTGAAGTTGGTCATTGGGTCTGCCGAGCAACTGGGTTATCCACCTCCACCACATTTAAATCGGTCGCCGATCGCGATACCTTGAAACTGACCGCGCGTGAATCAGAAGTCCTATTTCTGCTCTTATACGGTAAAACGGCCAAGGATATCGCTGGCATGCTGGGGCGCTCTCCCCGCACCATCGAACACGCTATCGAGCGCATCCGCAACAAATTCGGTGCTTTGAGCAAAGACCAACTGATTGACCTTGCTTTAGATCGGGGATTCGGCTCAGTCATCCCGAAAACCTTGCTTAGAAAACAACTTTCCGTTGTGCTGAGCGATCACACGATCCCCAAAAAAGTTGACGTTGTCGCCCAGTAAGCAACAACGTCAAGCTGATTG | *vqmA_Vc_ *171*-*199* construction |
| ODgBlock-8 | GCAAGATTTAACCGACACTGCGATTCTTGAAGTTGGTCATTGGGTCTGCCGAGCAACTGGGTTATCCACCTCCACCACATTTAAATCGGTCGCCGATCGCGATACCTTGAAACTGACCGCGCGTGAATCAGAAGTCCTATTTCTGCTCTTATACGGTAAAAAACCGCAGCACATTGCACGAGTAATGGGGATTTCGATTAAAACGGTGGAAGGGTATGAGGCCAAACTACGCAGTAAATTTGGGGCAGGCAACAAGCGGGAGCTCATCGATATGGCCATGTCCAAGGGTTATTACAGCATGGTGCCAAAAGCCCTGTTTCACAAACAACTTTCCGTTGTGCTGAGCGATCACACGATCCCCAAAAAAGTTGACGTTGTCGCCCAGTAAGCAACAACGTCAAGCTGATTG | *vqmA_Vc_ *200*-*224* construction |
| ODgBlock-9 | GCAAGATTTAACCGACACTGCGATTCTTGAAGTTGGTCATTGGGTCTGCCGAGCAACTGGGTTATCCACCTCCACCACATTTAAATCGGTCGCCGATCGCGATACCTTGAAACTGACCGCGCGTGAATCAGAAGTCCTATTTCTGCTCTTATACGGTAAAAAACCGCAGCACATTGCACGAGTAATGGGGATTTCGATTAAAACGGTGGAAGGGTATGAGGCCAAACTACGCAGTAAATTTGGGGCATTGAGCAAAGACCAACTGATTGACCTTGCTTTAGATCGGGGATTCGGCTCAGTCATCCCGAAAACCTTGCTTAGAACACAGGTCTCGATGCTGCTCAAGTAGTAAGCAACAACGTCAAGCTGATTG | *vqmA_Vc_ *225*-*246* construction |
| ODgBlock-10 | GCAAGATTTAACCGACACTGCGATTCTTGAAGTTGGTCATTGGGTCTGCCGAGCAACTGGGTTATCCACCTCCACCACATTTAAATCGGTCGCCGATGTGGTCGGTCTCAACTTGACCGAACGCGAGGAACTGGTGCTGTTCTTCCTGCTTCGTGGCCGAACGGCCAAGGATATCGCTGGCATGCTGGGGCGCTCTCCCCGCACCATCGAACACGCTATCGAGCGCATCCGCAACAAATTCGGTGCTTTGAGCAAAGACCAACTGATTGACCTTGCTTTAGATCGGGGATTCGGCTCAGTCATCCCGAAAACCTTGCTTAGAAAACAACTTTCCGTTGTGCTGAGCGATCACACGATCCCCAAAAAAGTTGACGTTGTCGCCCAGTAAGCAACAACGTCAAGCTGATTG | *vqmA_Vc_ *150*-*199* construction |
| ODgBlock-11 | GCAAGATTTAACCGACACTGCGATTCTTGAAGTTGGTCATTGGGTCTGCCGAGCAACTGGGTTATCCACCTCCACCACATTTAAATCGGTCGCCGATCGCGATACCTTGAAACTGACCGCGCGTGAATCAGAAGTCCTATTTCTGCTCTTATACGGTAAAACGGCCAAGGATATCGCTGGCATGCTGGGGCGCTCTCCCCGCACCATCGAACACGCTATCGAGCGCATCCGCAACAAATTCGGTGCTGGCAACAAGCGGGAGCTCATCGATATGGCCATGTCCAAGGGTTATTACAGCATGGTGCCAAAAGCCCTGTTTCACAAACAACTTTCCGTTGTGCTGAGCGATCACACGATCCCCAAAAAAGTTGACGTTGTCGCCCAGTAAGCAACAACGTCAAGCTGATTG | *vqmA_Vc_ *171*-*224* construction |
