# Supplementary material for: Mechanism underlying the DNA-binding preferences of the Vibrio cholerae and vibriophage VP882 VqmA quorum-sensing receptors
Source: PLoS Genet. 2021 Jul 6;17(7):e1009550. doi: 10.1371/journal.pgen.1009550 (PMC8284805; doi:10.1371/journal.pgen.1009550)
Supplement: Attachment — Submitted filename: Duddy_ResponsetoReviewers.docx [file pgen.1009550.s016.docx]

Dear Dr. Crosson and Dr. Søgaard-Andersen,

Thank you for handling our manuscript.  We are grateful for the overall positive assessment of our work and for the insightful suggestions the reviewers provided. We have taken the reviewers’ comments to heart and revised the manuscript exactly as requested, including providing the new experiments suggested by the reviewers. Those changes have certainly improved the manuscript. We provide point-by-point responses to the reviewers’ comments below. Reviewers’ comments are in black text and our responses are in red text. We have uploaded a revised manuscript, revised supporting information, and source files. We hope you now find the manuscript acceptable for publication in *PLOS Genetics*.

Yours on behalf of all the authors,

Bonnie L. Bassler

**Response to Reviewer Comments**

*Reviewer #1:*

Duddy et al present a detailed mechanistic evaluation of the differences in function between two VqmA transcription factors: one from Vibrio cholerae that binds DPO and one encoded by phage VP882 that also binds DPO. This manuscript explores the amino acid differences between the VqmA proteins and the nucleic acid differences between the Pqtip and PvqmR binding sites that result in VqmAphage binding to both substrates but restrict VqmAVc to only binding PvqmR. Their conclusions are: 1) the 6 central nucleotides in the DNA binding sites drive promoter specificity, 2) VqmAphage and VqmAVc have specific DNA binding domains, whereas their PAS and dimerization domains are interchangeable, 3) two regions of VqmA are required to interact specifically with Pqtip. This was a really interesting read on a relevant topic. The experimental design was very clear and impressive in dissecting the mechanism of VqmA DNA binding that differs between the bacterium and phage. Overall, this was a clearly and expertly written paper. There are some wording choices that are not always substantiated with direct evidence (i.e., lack of quantified EMSAs, statistical analyses, etc.).

In addition, the authors should be aware that another publication recently came out that overlaps with some of the experiments in this manuscript:

Understanding the mechanism of asymmetric gene regulation determined by the VqmA of vibriophage.
Gu Y, Zhi SX, Yang N, Yang WS.
Biochem Biophys Res Commun. 2021 Apr 22;558:51-56. doi: 10.1016/j.bbrc.2021.04.036. Online ahead of print.
PMID: 33895551

Experiments that overlap:
• Chimeras with PAS domain swaps
• Modulation of Pqtip DNA binding site

Gu et al. also report a crystal structure of VqmAPhage-DPO. This will aid the authors in mapping the substituted VqmAphage residues from the random mutagenesis screen.

We thank the referee for the encouraging comments and the suggestions to improve our work. Indeed, while our present work was under review at *PLOS Genetics*, a newly reported crystal structure of the DPO-VqmA_Phage_ complex was published by Gu *et al.* (2021). The new structure suggests that the two DBDs in the VqmA_Phage_ dimer are more relaxed than those of the VqmA_Vc_ dimer. Gu *et al*. (2021) also identified a 9-nucleotide palindrome in P*qtip* that VqmA_Phage_ binds. As described below, under the relevant points for each referee, these two findings greatly inform our interpretations of our results. We now cite the manuscript by Gu *et al*. (2021) several times, we describe how their findings are consistent with ours, and most importantly, we use their new findings to deepen the interpretations we make concerning our results. We hope the referee enjoys the expanded insights we now report in the revised paper.

Major comments:

• Line 91 and 286 – “VqmAVC must possess an additional feature that prevents it from binding Pqtip”; couldn’t VqmAPhage possess the additional feature and VqmAVC be lacking said feature? The authors repeatedly say this isn’t the case, but how have the authors excluded that possibility? Which data specifically show that VqmAphage doesn’t have the additional feature that enables recognition of the 6 core nucleotides? Given the findings in the new study by Gu et al., the X-ray crystal structure indicates a more relaxed conformation on binding with DPO. Perhaps this is the feature that enables recognition?

We agree with the reviewer that the relaxed conformation of VqmA_Phage_ revealed by the newly reported DPO-VqmA_Phage_ crystal structure could be the “additional feature” that enables recognition of both P*vqmR* and P*qtip*.

As requested, we have modified lines 91, lines 286 and the paragraph in lines 346-360 to soften our interpretation and jointly address this possibility and the new VqmA_Phage_ crystal structure.

Line 91 now reads:

“Isolation of VqmA_Phage_ mutants capable of activating *vqmR* expression but incapable of activating *qtip* expression revealed conserved or functionally conserved residues in VqmA_Phage_ and VqmA_Vc_, indicating that VqmA_Vc_ likely possesses an additional feature that prevents it from binding P*qtip* DNA.”

Lines 282-287 now read:

“1) There are at least four residues (G201, A202, E207, and M211) required for VqmA_Phage_ to recognize P*qtip* DNA. 2) Because the VqmA_Phage_ G201D, G201R, A202V, E207K, E207V, and M211K variants exhibit WT binding to P*vqmR*, the substitutions at these four residues must not significantly affect P*vqmR* recognition. 3) Because these residues are conserved or similar between VqmA_Phage_ and VqmA_Vc_, one would expect VqmA_Vc_ to have the capacity to bind P*qtip*. 4) However, because VqmA_Vc_ in fact does not bind P*qtip*, VqmA_Vc_ likely possesses an additional feature that resides elsewhere in the protein that prevents P*qtip* binding from occurring.”

The paragraph in 346-360 now reads:

“The main goal of the present work was to discover features of the VqmA proteins that confer specificity in transcriptional activity. We propose that phage VP882 VqmA_Phage_ possesses a feature that relaxes its DNA-binding specificity and *V. cholerae* VqmA_Vc_ possesses a feature that restricts its DNA-binding capability. Regarding VqmA_Vc_, our genetic analyses support the hypothesis that the VqmA_Vc_ DBD harbors elements that prevent it from binding P*qtip*. This hypothesis stems from our finding that residues G201, A202, E207, and M211 are crucial for VqmA_Phage_ recognition of P*qtip*. These residues are conserved between VqmA_Vc_ and VqmA_Phage_. Specifically, in VqmA_Vc_ they are: G199, A200, Q205, and L209, respectively. More broadly, sequence alignments of VqmA proteins among *Vibrios* reveal that the residue at the 207^th^ position in VqmA_Phage_ (205^th^ position in VqmA_Vc_) is most frequently either a Glu or a Gln (Silpe *et al.* 2019a). Similarly, the residue at the 211^th^ position in VqmA_Phage_ (209^th^ position in VqmA_Vc_) is commonly a hydrophobic residue, like Met, Leu, Ile, or Val. Thus, E207 and M211 are not unique to VqmA_Phage_, but rather occur in most VqmA proteins. We propose that because the key residues for P*qtip* binding are conserved in VqmA_Phage_, VqmA_Vc_, and other *Vibrio* VqmA proteins, VqmA_Vc_ is likely restricted from binding P*qtip* by additional features elsewhere in its DBD. Regarding VqmA_Phage_, the DPO-VqmA_Phage_ structure was reported during review of this manuscript (Gu *et al.* 2021). Superimposition of this new structure (7DWM) onto the DPO-VqmA_Vc_ and DPO-VqmA_Vc_-P*vqmR* structures (6KJU and 6IDE, respectively, and Wu *et al.* 2019a, Wu *et al.* 2019b, Huang *et al.* 2020) reveals two insights (Fig. S8). First, the conformations of the three PAS domains are similar except for the orientations of the first 20 N-terminal residues in each protein, indicating that the PAS domains do not confer the differences in promoter DNA specificity. Second, the DPO-VqmA_Phage_ DBDs adopt a conformation that is intermediate between that of the more open DBDs in the DPO-VqmA_Vc_ structure and the closed DBDs in the DPO-VqmA_Vc_-P*vqmR* structure. Additionally, the interaction interface between the VqmA_Phage_ DBDs is less extensive, and thus more relaxed than that of the VqmA_Vc_ DBDs (Gu *et al.* 2021). Likely, the more relaxed conformation exhibited by the VqmA_Phage_ DBDs underpins its promiscuity for promoter binding with respect to P*vqmR* and P*qtip*.”

• Line 138-139 - “the respective DBD of each purified VqmA protein drives promoter selectivity.”; The C-terminal swap does not appear to be the only driver of promoter selectivity here as can be seen with the “~7-fold lower affinity”. Is the 7-fold lower affinity referring to the lux results? First, I think quantifying these shifts (in triplicate) would really help here with comparing binding of these chimeras. Second, is some sort of additional allostery at play here (besides the DPO ligand-dependent dimerization)? The dimerization experiments do indeed show that promoter-binding selectivity is mostly mediated by the DBD, but I do not believe it is “governed exclusively by the DBD” as mentioned on Line 168. This claim seems too strong, especially given the data shown in later figures. The decreases in DNA binding when using the GST dimerization method shows it cannot just be the DBD, at least not in the context shown here. I suggest that the authors either weaken wording or substantiate their claim with quantified EMSA binding data.

We thank the referee for these good suggestions.

The “7-fold lower affinity” reported for _Phage_N-C_Vc_ relative to WT VqmA_Vc_ did refer to the EMSA data, not the *lux* results. We have clarified exactly how we arrived at this number in the text. In line 130, we now write:

“Our EMSA analyses show that _Phage_N-C_Vc_, like VqmA_Vc_, only bound P*vqmR*, but with an estimated ~7-fold lower affinity.”

We regret the confusion, but we had indeed quantified the DNA shifts for the WT and VqmA chimeric proteins from multiple EMSAs identical to the representative ones shown in Fig. 1 to obtain the estimated EC_50_ values. The quantifications are provided in Fig. S2. We have accordingly adjusted the Fig. S2 legend to clarify this issue.

As suggested by the referee, we have removed the word “exclusively” in line 24 of the Abstract, and line 168 of the Results.

Regarding allostery: We do not know at this point. What we do know is that VqmA dimerization does not depend on binding to DPO as apo-VqmA purifies as a dimer. A conformational change occurs upon ligand binding as limited-proteolysis patterns of apo-VqmA and DPO-VqmA differ (Huang *et al.* 2020). Additional allosteric-driven changes in activity remain a possibility.

• Line 186 – “VqmAVC only shifted Pqtip*”; this protein still appears to have a slight shift of PvqmR at the two highest concentrations (300 and 600 nM I believe). There is a distinct band in the bound region for the 300 nM condition. Change wording or consider repeating EMSAs with BSA or other protein to decrease non-specific binding, if that is indeed what is happening in that gel shift.

As requested, we have changed the wording in line 186 to:

“VqmA_Vc_ shifted P*qtip**, whereas it only weakly shifted P*vqmR**.”

As requested, we examined the binding of VqmA_Vc_ and VqmA_Phage_ to P*vqmR** and P*qtip** in the presence of BSA. The results were nearly identical in the presence and absence of BSA, and so they do not change our conclusion. We have included the BSA results here for the referee.

• Line 192-193 – consider replacing “is sufficient to prevent VqmAVC from binding” to a more accurate statement based on the EMSAs and transcriptional fusion assays, such as “decreases VqmAVC binding affinity” or “prevents VqmAVC transcription at Pqtip.”

As requested, we changed the sentence in line 192-193 to say:

“Moreover, the presence of the -AAAATA- nucleotide sequence in P*qtip* is sufficient to prevent VqmA_Vc_ from activating transcription of P*qtip*.”

• Line 213-214 – It does not appear that all four VqmAPhage single point mutants possessed WT activity (unless after statistical analysis it can be shown that there is no significant difference between these activities.)

We thank the referee for the good suggestion. We have now included statistical analyses in the bar graphs in Fig. 3 to substantiate our results. In the case of the data in Fig. 3B, the statistics show that the activity of the four VqmA_Phage_ point mutants differ significantly from WT VqmA_Phage_, albeit by less than 2-fold. We modified our statement in lines 213-214 to say:

“Analysis of P*vqmR-lux* and P*qtip-lux* expression revealed that all four VqmA_Phage_ single point mutants possessed levels of activity within 2-fold of that of WT P*vqmR-lux* …”

• Figures 3B, 3C, 3E, and 3F would benefit from statistical analysis to help solidify some of the claims made in the text.

As requested, statistical analyses have been added to these graphs. The figure legend has been modified to describe the statistical methods.

• Lines 282-287: The section discussing the conclusions of the random mutagenesis of the VqmAphage DBD is reaching. Point 2 stating that the four residues in VqmAphage must not play any role cannot be stated from these data. Only a couple substitutions were tested, and one could argue that the lack of a bulky or charged amino acid in place of the existing amino acids is “playing a role”. Point 3: why do the authors state that VqmAVc has the capacity to bind Pqtip? I understand that analogous amino acids exist in VqmAphage, but at this point there are no defined data showing that it has the ability to bind Pqtip. This statement is odd, given that the protein itself doesn’t bind Pqtip, which the authors have shown experimentally.

As noted in the response to the referee’s first point, we changed the wording in lines 282-287 to clarify our interpretation and to be more accurate (copied from above):

“1) There are at least four residues (G201, A202, E207, and M211) required for VqmA_Phage_ to recognize P*qtip* DNA. 2) Because the VqmA_Phage_ G201D, G201R, A202V, E207K, E207V, and M211K variants exhibit WT binding to P*vqmR*, the substitutions at these four residues must not significantly affect P*vqmR* recognition. 3) Because these residues are conserved or similar between VqmA_Phage_ and VqmA_Vc_, one would expect VqmA_Vc_ to have the capacity to bind P*qtip*. 4) However, because VqmA_Vc_ in fact does not bind P*qtip*, VqmA_Vc_ likely possesses an additional feature that resides elsewhere in the protein that prevents P*qtip* binding from occurring.”

We also changed our wording in lines 358-360 in the Discussion (copied from above):

“We propose that because the key residues for P*qtip* binding are conserved in VqmA_Phage_, VqmA_Vc_, and other *Vibrio* VqmA proteins, VqmA_Vc_ is likely restricted from binding P*qtip* by additional features elsewhere in its DBD.”

• It would help for the authors to discuss more details about the failed mutagenesis in which VqmAVc mutants were not able to activate Pqtip. How many mutants did they screen? What was the average mutagenesis rate per allele? Of course, it is possible that by random mutagenesis the number of substitutions required to enable VqmAVc to bind Pqtip may never work. But this is hard to assess without more details.

As requested, we modified the existing text in lines 293-297 to now say:

“To do this, we constructed a library of random *vqmA_Vc_* DBD alleles containing, on average, 1-2 substitutions, and we cloned them into a plasmid under an arabinose-inducible promoter. The library was transformed into the ∆*tdh* *E. coli* strain harboring the P*qtip-lux* reporter and transformants were propagated on plates containing arabinose. We screened ~10,000 transformants for colonies that produced light indicating that they contained VqmA_Vc_ proteins that activated P*qtip-lux*.”

• The authors nicely show that the 6 core nucleotides drive specificity – great experiment! But the authors don’t comment on the residues shown to contact these bases in the DNA-bound VqmAVc structure: Gln174, Lys172, Lys203, Glu188, Arg195, Lys185, Tyr186. The VqmAVc171-199 mosaic should have these all. Could the authors comment on this inconsistency in these two results?

We thank the referee for the positive comment about this experiment. As the referee noted, according to the DPO-VqmA_Vc_-P*vqmR* structure, Gln174, Lys172, Lys203, Glu188, Arg195, Lys185, Tyr186 interact with the nucleotide bases, not the DNA backbone of the 6 core nucleotides in P*vqmR* (Wu *et al.* 2019a). This observation is fully consistent with our result that replacing the 6 nucleotide -TTTCCC- stretch in P*vqmR* with the corresponding -AAAATA- nucleotides from P*qtip*, makes VqmA_Vc_ fail to shift P*vqmR**. To address why the VqmA_Vc_^*171-199^ mosaic does not switch preferences following the promoter fragment exchange: We suggest in the Discussion that a particular regional conformation or local environment might be generated by amino acids that are not contiguous in linear sequence but are in close proximity in 3D, and that arrangement is necessary for VqmA proteins to bind DNA. Whatever the necessary conformation/local environment is, we speculate that it does not form in the in VqmA_Vc_^*171-199^ mosaic.

Minor comments:
• Line 33: “as” necessary sounded awkward…perhaps “that is”

We have made the change as requested.

• Line 50: if VqmAvc is overexpressed…will it bind the phage BS?

VqmA_Vc_ overexpression does not enable binding to P*qtip*. In all of our *in vivo* experiments, we control VqmA protein production via administration of the arabinose inducer. At saturating levels of arabinose (0.2%), VqmA_Vc,_ although overproduced, remains unable to activate P*qtip-lux* expression or induce phage lysis (Fig. 1B and 1C).

• Line 273: specify that it is PvqmR DNA in the structure.

As requested, we have exchanged “DPO-VqmA_Vc_­-DNA” with “DPO-VqmA_Vc_-P*vqmR*” in line 273, and throughout the text.

*Reviewer #2:*

Bassler and coworkers have previously investigated the behavior of a prophage-encoded gene, VqmAphage, a homolog of a chromosomally encoded gene (VqmA) in Vibrio cholerae. Both genes are transcriptional activators that become active in the presence of the quorum-sensing signal DPO; the phage gene turns on an anti-repressor, leading to phage induction and lysis of its host. Here, the authors investigate the basis for asymmetric binding and activity of these two proteins; while both activate the host target gene vqmR, only the phage gene can act on the phage target (qtip). Via chimeric targets and proteins, the authors demonstrate specificity determinants in both target and protein, and also find that the bacterial VqmA contains multiple sequences that interfere with its ability to activate the prophage promoter. The work provides a range of insights into specificity determinants, although, as noted, actually understanding how these determinants work will likely require structures.

1. In terms of the logic of the regulatory circuit, it seems reasonable that the host gene cannot activate phage lysis. When, however, would it be particularly useful for the phage, as it starts into a lytic cycle, to activate the host vqmR gene?

The referee brings up an excellent point about which we, too, are especially curious. We do not yet understand how manipulation of host quorum sensing benefits the phage in natural settings. We hope that our continued exploration of mechanisms underlying phage components, including our analysis of the VqmA DNA-binding asymmetry, will provide insight and valuable tools to probe this central question.

2. Line 79-81: This idea that it is surprising a regulator “can recognize …promoters… present in genes in different kingdoms” seems like over-selling this story. After all, while one can say the virus is in a different kingdom, it is entirely dependent on the bacteria for growth, and phage (and animal viruses) clearly manipulate host machinery in many ways.

As requested, we have softened the language. In lines 79-81 we now write:

“Thus, how VqmA_Phage_ can recognize two different promoters, while VqmA_Vc_ cannot, is not understood.”

3. Introduction, lines 82-98: This reiteration of everything in the paper is much more detailed then is needed here, and as a result doesn’t emphasize the most important points. Why list all the specific residues here?

We agree and we thank the referee for pointing out the redundancy. We have removed the details about specific residues in lines 87-92 of the Introduction.

4. Lines 110-117: Please clarify why these experiments were all done in the absence of DPO. I did not understand the explanation offered here. DPO is not needed for DNA binding? This is implied, but not said. Is the activation being measured in this paper different in other requirements to what is seen with DPO? Or does that simplify amplify signals, and overproducing the proteins under arabinose induction leads to the same response? In particular, given the focus here, is the mode of activation the same?

We agree with the referee that we can be more explicit here. In lines 113-114 we now write:

“Apo-VqmA displays basal transcriptional activity *in vivo* (Huang *et al.* 2020). Thus, while DPO enhances VqmA DNA-binding activity, it is not an absolute requirement for binding.”

As the referee correctly assumed, VqmA DNA-binding activity can be amplified either by over-production of VqmA proteins or by administration of DPO. In our experiments, by assaying DNA-binding in the absence of DPO, we ensure that we are exclusively examining VqmA DNA-binding function as opposed to any DPO-driven enhancement of VqmA DNA-binding.

5. P. 7, lines 140-147: The question about the PAS domain playing a role in promoter binding selectivity may be reasonable, but given that removing it renders the proteins entirely inactive, the way this is set up is not useful. I would suggest starting with the question posed more generally, or just a simple statement that deleting the PAS domain led to inactive proteins. Then the suggestion that this might be because of a requirement for dimerization can be made and tested. I’m not convinced by the GST results that one can rule out a role for PAS, given the authors’ finding later that sequences elsewhere in the protein have a restrictive effect on DNA binding patterns.

We thank the referee for this good suggestion. We have made the changes to lines 140-147 as requested. The text now reads:

“We next assayed the VqmA_Vc_ and VqmA_Phage_ DBDs lacking their PAS domains (DBD_Vc_ and DBD_Phage_, respectively) for activation of P*vqmR-lux* and P*qtip-lux*. Deletion of the PAS domains resulted in inactive proteins as neither DBD activated transcription (Fig. S3A and S3B, respectively), and likewise, EMSA analyses showed that neither DBD bound either promoter (Fig. S3C). Gel filtration analyses indicated that the DBD proteins purified as monomers (Fig. S3D), suggesting that the DBDs were unable to dimerize in the absence of their partner PAS domains. This result is consistent with previous findings that, in addition to sensing DPO, the VqmA_Vc_ PAS domain is responsible for dimerization (Wu *et al.* 2019a, Huang *et al.* 2020).”

We also made changes to lines 166-168 as follows:

“Taken together, our results show that VqmA promoter-binding selectivity is conferred by the DBD, and that dimerization is necessary.”

6. The demonstration that a change of a central region in the VqmA binding region form AAAATA to TTTCCC is striking and could be further investigated. For instance, this result might suggest that the angle/bending between the dimer binding sites for VqmA plays an important role in the specificity of binding of the different proteins. Was this looked at all? Would changing spacing even slightly change binding specificity? It would be useful to include in Figure 2 where the start point for transcription is, where -10, -35 sequences are. If this is not relevant (see query 4 above), please provide a clear explanation of why not.

We concur that the identification of this site is a key finding of our work, but we did not test changing the spacing between dimer binding sites. We also agree that the distance and/or angle between the binding sites is important for specificity. While our manuscript was under review, work by another group was published that also investigated the mechanism of VqmA_Phage_ specificity for P*qtip* (Gu *et al.* 2021). Consistent with our analyses, these authors identified the same key sequence in P*qtip*. Importantly, their results indicate that VqmA_Phage_ can recognize a longer palindromic sequence (GG-N_9_-CC) compared to VqmA_Vc_ (GG-N_6_-CC). This finding provides us a mechanism to address the referee’s questions. We do so in a new paragraph of our Discussion:

“Previous work demonstrated that VqmA_Vc_ recognizes a key GG-N_6_-CC palindrome in P*vqmR* (Papenfort *et al.* 2015, Wu *et al.* 2019a). Our sequence alignment of P*vqmR* and P*qtip* showed that P*qtip* does not possess this palindrome. Rather, the corresponding sequence in P*qtip* is GG-N_6_-TA (Fig. 2A). The most obvious divergence between the two sequences is in the central six nucleotides: “-AAAATA-” in P*qtip* and “-TTTCCC-” in P*vqmR* (Fig. 2A). We hypothesized that this nucleotide stretch could be responsible for conferring the asymmetric DNA-binding patterns to the two VqmA proteins. Indeed, exchanging these nucleotides in P*qtip* and P*vqmR* reversed the promoter binding preferences of the VqmA proteins. We verified our conclusion that this core 6 nucleotide stretch drives VqmA-DNA binding preference using our VqmA chimeric proteins (_Vc_N-C_Phage_ and _Phage_N-C_Vc_), a representative mosaic protein (VqmA_Vc_^*171-224^), and a representative protein containing a point mutation (VqmA_Phage_^G201D^) (Figs. S9A and S9B and S10). While the present manuscript was under review, Gu *et al.* (2021) reported that a GG-N_9_-CC palindrome in P*qtip* is the key sequence for VqmA_Phage_ recognition. According to our DNA sequence alignment, the GG-N_6_-CC palindrome required for VqmA_Vc_ binding is only present in P*vqmR*, while the key GG-N_9_-CC palindrome required for VqmA_Phage_ binding exists in both P*qtip* and P*vqmR* (Fig. 2A). Together, our results and those of Gu *et. al.* (2021) explain, at the level of the promoter DNA, why VqmA_Phage_ binds both P*qtip* and P*vqmR* while VqmA_Vc_ recognizes only P*vqmR.*”

As suggested, we have labeled the transcriptional start sites in Fig. 2.

7. Do the various chimeras and mutants all show the same discrimination based on the central region of the binding site? Is this the only specificity determinant in the promoter? Mutant proteins might help to determine that; certainly in Fig. 2C, D, changing the site doesn’t totally lead to VqmAVC acting like the phage protein on pqtip, just relieves the stringency modestly.

We thank the referee for the excellent suggestions. We have now tested the abilities of the _Vc_N-C_Phage_ and _Phage_N-C_Vc_ chimeras, the VqmA_Vc_^*171-224^ mosaic, and the VqmA_Phage_^G201D^ mutant to bind P*vqmR** and P*qtip**. The results are all consistent: The VqmA DNA-binding preferences for all of these proteins were inverted by exchanging the core 6 nucleotides in P*vqmR* and P*qtip*. We have included these new data in new Figs. S9A and S9B and S10. As noted in our response to the preceding comment, we added the following sentences:

“We verified our conclusion that this core 6 nucleotide stretch drives VqmA-DNA binding preference using our VqmA chimeric proteins (_Vc_N-C_Phage_ and _Phage_N-C_Vc_), a representative mosaic protein (VqmA_Vc_^*171-224^), and a representative protein containing a point mutation (VqmA_Phage_^G201D^) (Figs. S9A and S9B and S10).”

We also added the following new passages to the Discussion based on the referee’s suggestions (again, copied from above):

“Regarding VqmA_Phage_, the DPO-VqmA_Phage_ structure was reported during review of this manuscript (Gu *et al.* 2021). Superimposition of this new structure (7DWM) onto the DPO-VqmA_Vc_ and DPO-VqmA_Vc_-P*vqmR* structures (6KJU and 6IDE, respectively, and Wu *et al.* 2019a, Wu *et al.* 2019b, Huang *et al.* 2020) reveals two insights (Fig. S8). First, the conformations of the three PAS domains are similar except for the orientations of the most N-terminal 20 residues in each protein, indicating that the PAS domains do not confer the differences in promoter DNA specificity. Second, the DPO-VqmA_Phage_ DBDs adopt a conformation that is intermediate between that of the more open DBDs in the DPO-VqmA_Vc_ structure and the closed DBDs in the DPO-VqmA_Vc_-P*vqmR* structure. Additionally, the interaction interface between the VqmA_Phage_ DBDs is less extensive, and thus more relaxed than that of the VqmA_Vc_ DBDs (Gu *et al.* 2021). Likely, the more relaxed conformation exhibited by the VqmA_Phage_ DBDs underpins its promiscuity for promoter binding with respect to P*vqmR* and P*qtip*.”

8. Fig. 3B, C, Line 215: The authors focus here on E194A as the least active on the qtip promoter but R164I looks equally defective in the promoter activity assay. The basis for concluding (as in line 220-221) that only E194 matters is therefore only due to its less extreme lysis effect? The fact that these don’t fully correlate might suggest an addition role for VqmAphage in the lysis pathway; has that been ruled out?

This is an interesting point. At present, we do not have a method to distinguish whether differences in the VqmA_Phage_ R184I and the E194A mutant phenotypes in the lysis and transcriptional reporter assays are a consequence of using a recombinant *E. coli* system or whether they point to some interesting biology. Regarding an additional role for VqmA_Phage_ in the lysis pathway, we have no evidence for that. We previously screened for phage components that function downstream of VqmA_Phage_ in the lytic program (Silpe *et al.* 2019a). The only gene we discovered directly controlled by VqmA_Phage_ was *qtip*.

9. Line 248: clarify that the mutation library is in the context of the otherwise full-length protein.

As requested, we changed line 248 to say:

“We constructed a library of random mutations in the region of *vqmA_Phage_* encoding the DBD in the context of the full-length gene...”

10. Line 282-287: The conclusions reached here need more explanation and could be rewritten. Was the mutagenesis and selection/screening method for finding these four residues saturating? The first two conclusions follow from the way these were identified, requiring that they still activate vpmR; maybe make that more explicit here? Where does the conclusion that both proteins “possess the capacity to bind pqtip DNA” come from? That these residues are identical in VqmA? Why is this evidence of the capacity to bind? Maybe less definitive conclusions and a suggestion that because mutations which interfere with qtip binding by VqmAphage are not in residues only found in the phage protein, likely specificity lies elsewhere. Then the suggestion that this is in the restriction of VqmA binding, rather than (or in addition to) positive elements in VqmAphage.

The selection and screen were not saturating. Other residues in the VqmA_Phage_ 201-211 region could be important for P*qtip* recognition. As requested, we changed the wording in lines 282-287 to be more accurate and to clarify that one would “expect” VqmA_Vc_ to bind P*qtip* but it does not. The revised paragraph reads:

“1) There are at least four residues (G201, A202, E207, and M211) required for VqmA_Phage_ to recognize P*qtip* DNA. 2) Because the VqmA_Phage_ G201D, G201R, A202V, E207K, E207V, and M211K variants exhibit WT binding to P*vqmR*, the substitutions at these four residues must not significantly affect P*vqmR* recognition. 3) Because these residues are conserved or similar between VqmA_Phage_ and VqmA_Vc_, one would expect VqmA_Vc_ to have the capacity to bind P*qtip*. 4) However, because VqmA_Vc_ in fact does not bind P*qtip*, VqmA_Vc_ likely possesses an additional feature that resides elsewhere in the protein that prevents P*qtip* binding from occurring.”

11. I’m assuming that this 171-224 construct still has the rest of the sequence past 224 to the end of VqmA. Is that correct? Was a simple truncation of the C-terminal amino acids, maybe ending with the K found in VqmAphage, ever tested? How does the +171-224 VqmA construct act on the slightly permissive DNA binding site chimera (pqtip*)?

Yes, that is correct. The VqmA_Vc_^*171-224^ construct harbors the residues in VqmA_Vc_ beyond the 224^th^ position. We did exchange the C-terminal residues beyond residue 224 in VqmA_Vc_ with the corresponding residues in VqmA_Phage_ (VqmA_Vc_^*225-246^). Since the VqmA_Phage_ protein is 13 residues shorter than VqmA_Vc_, this mosaic is effectively a VqmA_Vc_ truncation. VqmA_Vc_^*225-246^, like WT VqmA_Vc_, did not bind P*qtip* (see Fig. 5B), indicating that this region of the DBD does not play a role in promoter restriction. We did not test additional VqmA_Vc_ truncations since VqmA_Vc_^*225-246^ did not exhibit an interesting phenotype.

As noted above, we tested the ability of the VqmA_Vc_^*171-224^ mosaic to bind P*vqmR*, P*qtip*, P*vqmR** and P*qtip** in EMSAs. The VqmA_Vc_^*171-224^ mosaic protein shifted all four probes, which is consistent with our results that, like VqmA_Phage_, VqmA_Vc_^*171-224^ is promiscuous. We have included these new data in a new Fig. S10A and S10B.

12. Discussion: Wouldn’t it be fairly easy to see if removing hns leads to more permissive VqmA(phage mutants or host) binding/activity?

We thank the referee for this good idea. In response, we performed experiments to assess the ability of the WT and mutant VqmA_Phage_ proteins to outcompete H-NS for binding to P*qtip in vitro*. Our results show that there was no difference between WT VqmA_Phage_ and VqmA_Phage_^G201D^ binding to P*qtip* DNA in the presence of purified H-NS. These experiments eliminate the possibility that H-NS competition underlies our findings. We have included these data in new Fig. S9C and S9D.

Additionally, we removed the sentence in lines 390-391:

“Any potential role for H-NS in transcriptional regulation of *vqmR* or *qtip* has not been investigated.”

We replaced it with:

“To address this possibility, we examined whether WT VqmA_Phage_ and VqmA_Phage_^G201D^ competed with H-NS for binding to P*qtip* using EMSA assays. There was no difference between WT VqmA_Phage_ and VqmA_Phage_^G201D^ binding to P*qtip* DNA in the presence of purified H-NS (Fig. S9C and S9D). These experiments eliminate the possibility that H-NS competition underlies our findings.”

13. Discussion: Is there any cost to the host of expressing the VqmAphage in cells not carrying a prophage? Would that help to address whether there is any other promiscuous activity of this protein?

This is a really fascinating point. We do not know yet! All we know is that expression of *vqmR* inhibits biofilm formation in *V. cholerae* (Papenfort *et al.* 2015, Papenfort *et al.* 2017). We hope to focus on such questions in future work.

14. Figure 1 D, G, elsewhere: I found it somewhat confusing to have the triangles indicating increasing amounts of VqmA protein labeled with the DNA it is binding to. Maybe put the pvqmR, etc. labels below the gel, just have the relevant protein above?

We appreciate this suggestion. We have made the requested changes to all relevant figures. They are less busy/easier to follow now.

*Reviewer #3:*

In the study " Mechanism underlying the DNA-binding preferences of the Vibrio cholerae and vibriophage VP882 VqmA quorum-sensing receptors", the Dr Bassler’s group use a mutagenic approach to pinpoint the molecular determinants of VqmA transcriptional factor selectivity. VqmA transcriptional factor is a quorum-sensor receptor of DPO autoinducer that in Vibrio cholerae controls genes involved in biofilm formation and virulence factor production. Recently, this same group described that the phage VP882, which infect V. cholerae, also encodes for a VqmA receptor (VqmA-phage) that is similarly regulated by DPO. VqmA-phage controls the transcription of the phage gene qtip, which regulates lytic program, but it is also able to regulate the same genes that the bacterial host VqmA (VqmA-Vc)i regulates. In contrast, VqmA-Vc is not able to regulate qtip, thus showing a transcriptional asymmetry between both VqmA transcriptional factors that has very important biological implications. In the present manuscript, the authors attempt to reveal the molecular mechanism of this transcriptional asymmetry by generating a battery of mutants in both VqmA receptors (bacterial and phage) and analyzing their ability to bind to the VqmR (bacterial) and qtip (phage) promoters by EMSA or with reporter gene, as well as to induce the lytic cycle of phage VP822. These assays evaluate the contribution of some residues and/or protein fragments to the transcriptional asymmetry in order to reveal their molecular determinants. The work is very well executed, the results are clear, the manuscript is well written and the logic is easy to follow, as is usual for the manuscripts of this group. However, I consider that it fails in its main claim, since the results do not allow the authors to define the molecular mechanism of the asymmetry. The results show that certain residues contribute to this asymmetry and that it possibly required the synergistic contribution of different portions or elements of the receptor to confer this asymmetry, but these data are not sufficient to propose a molecular mechanism. In fact, several alternatives are proposed in the discussion, some of which may be more feasible, but in no case can the data presented clearly answer the main question, the molecular mechanism of VqmA asymmetry, As is claimed at different points throughout the manuscript (e.g. pag 3, line 50: ”we discover the molecular mechanism underpinning the asymmetric transcriptional preferences of the phage-encoded and bacteria-encoded VqmA proteins). In this sense, I consider that the authors do not address this problem by making use of all available information, much of it generated by themselves, on the molecular mechanisms of the recognition of operators by VqmA.

For VqmA-Vc, the molecular basis of VqmR operator recognition is quite well known since the three-dimensional structures of this protein bound to DPO (PDBs
6KJU and 6UGL, the last one solved by the manuscript’s authors) and, more importantly, VqmR operator (PDB 6IDE) have been reported. This last structure shows that VqmA adopts a quasi-symmetric dimeric form to recognize a GG palindromic sequence (GGN6CC) separated by 6 nucleotides (Wu et al, JBC 2019; PMID: 30610119). The direct readout of this sequence is performed by only 3 VqmA-Vc residues where a Lys (K185) has the major contribution. In fact, Bassler’s group showed in other manuscripts (Huang et al, JBC 2020; PMID: 31964715) that mutation of this Lys to Ala completely abolished the VqmA-Vc binding to its target DNA.

In the present manuscript the authors show that the qtip promoter does not retain the GGN6CC box since the second CC has been replaced by TA, but the two nucleotides following this TA are CC, so that the GG palindrome could be regenerated again, but separated now by 8 nucleotides (GGN8CC) in its promotor. The authors show in this manuscript that the exchange of the region between the GG palindrome in the VqmR and qtip operators is sufficient to change the asymmetry (first section of results “VqmA promoter-binding selectivity is conferred by the DNA-binding domain”) but, surprisingly, they do not elaborate further on this direction to explain the asymmetry. Clearly, this observation opens three options, i) VqmA-phage is able to read both GGN6CC and GGN6TA, although with different affinity, ii) VqmA-Vc reads GGN6CC and VqmA-phage reads GGN8CC, and iii) VqmA-phage has a higher plasticity than VqmA-Vc and can recognize GG palindrome with 6 and 8 nucleotide separation. Although I see the second option as more likely, I think the authors should have tested three possibilities if they want to determine the molecular basis of the asymmetry.

We thank the referee for providing detailed ideas concerning this set of manuscripts. With respect, we obviously disagree with the conclusion that our results are not sufficient to propose a mechanism. Our primary aim was to determine what features in the VqmA proteins and their target promoters drive their respective DNA-binding activities. Regarding mechanism from the protein side: Our genetic and biochemical analyses indicate that VqmA_Vc_ likely harbors a restrictive element in its DBD that prevents it from binding P*qtip*. The recent publication of the DPO-VqmA_Phage_ crystal structure revealed that the VqmA_Phage_ DBDs are more relaxed than those of VqmA_Vc_, which could promote recognition of two promoters (Gu *et al.* 2021). Our mosaic experiments show that differences in amino acids between residues 171-224 in VqmA_Vc_ and 173-226 in VqmA_Phage_ are likely responsible for both of these features. From the DNA side, our results with P*vqmR** and P*qtip** show that a core six nucleotides drive the different DNA-binding preferences of the two VqmA proteins. Indeed, the DPO-VqmA_Vc_-P*vqmR* structure and the recent publication of Gu *et. al.* (2021), together with our results, show that the key GG-N_6_-CC palindrome required for VqmA_Vc_ binding is only present in P*vqmR*, while the key GG-N_9_-CC palindrome required for VqmA_Phage_ binding exists in both P*qtip* and P*vqmR*. Thus, the findings by Gu *et al.* (2021) support our conclusions regarding mechanism from the DNA side. We hope the referee will agree that our studies have made real headway in defining how these two fascinating proteins function, and how they come to their distinct activities.

To address these issues and to incorporate the findings of Gu *et al.* (2021), we have added this new paragraph to the Discussion:

“Previous work demonstrated that VqmA_Vc_ recognizes a key GG-N_6_-CC palindrome in P*vqmR* (Papenfort *et al.* 2015, Wu *et al.* 2019a). Our sequence alignment of P*vqmR* and P*qtip* showed that P*qtip* does not possess this palindrome. Rather, the corresponding sequence in P*qtip* is GG-N_6_-TA (Fig. 2A). The most obvious divergence between the two sequences is in the central six nucleotides: “-AAAATA-” in P*qtip* and “-TTTCCC-” in P*vqmR* (Fig. 2A). We hypothesized that this nucleotide stretch could be responsible for conferring the asymmetric DNA-binding patterns to the two VqmA proteins. Indeed, exchanging these nucleotides in P*qtip* and P*vqmR* reversed the promoter binding preferences of the VqmA proteins. We verified our conclusion that this core 6 nucleotide stretch drives VqmA-DNA binding preference using our VqmA chimeric proteins (_Vc_N-C_Phage_ and _Phage_N-C_Vc_), a representative mosaic protein (VqmA_Vc_^*171-224^), and a representative protein containing a point mutation (VqmA_Phage_^G201D^) (Figs. S9A and S9B and S10). While the present manuscript was under review, Gu *et al.* (2021) reported that a GG-N_9_-CC palindrome in P*qtip* is the key sequence for VqmA_Phage_ recognition. According to our DNA sequence alignment, the GG-N_6_-CC palindrome required for VqmA_Vc_ binding is only present in P*vqmR*, while the key GG-N_9_-CC palindrome required for VqmA_Phage_ binding exists in both P*qtip* and P*vqmR* (Fig. 2A). Together, our results and those of Gu *et. al.* (2021) explain, at the level of the promoter DNA, why VqmA_Phage_ binds both P*qtip* and P*vqmR* while VqmA_Vc_ recognizes only P*vqmR.*”

Options 1 and 2 are easily analyzable and in fact the authors have already developed some of the tools to do it. The sequence alignments show that the Lys185 position at VqmA-Vc is occupied by an Arg in VqmA-phage, although both residues are similar in nature it is possible that this change may allow VqmA-phage to be more promiscuous and recognize both GGN6CC and GGN6TA or read alternatively GG palindrome separated by N6 o N8. The evaluation of this position should be a key point for understand the asymmetry.

The referee provides an interesting suggestion. In response, we constructed the requested two new mutants, VqmA_Vc_^K185R^ and VqmA_Phage_^R187K^ and we tested their abilities to activate P*vqmR-lux* and P*qtip-lux* in our recombinant *E. coli* system. Our results are shown below. The experiments were conducted exactly as in the manuscript (Black, no arabinose; white, 0.2% arabinose). The activity of each mutant mimicked that of the respective WT protein: VqmA_Vc_^K185R^ only activated P*vqmR-lux*, whereas VqmA_Phage_^R187K^ activated both P*vqmR-lux* and P*qtip-lux*. These new results demonstrate that substitution of Lys with Arg at the 185^th^ position in VqmA_Vc_ does not confer DNA-binding promiscuity, neither by recognition of GG-N_6_-TA nor the longer GG-N_9_-CC palindrome. Similarly, substitution of Arg with Lys at the corresponding position in VqmA_Phage_ does not abrogate DNA-binding promiscuity suggesting they do not play roles in driving asymmetric VqmA DNA-binding activities.

The second option is also easy to evaluate by carried out identical assays to those presented in the manuscript (EMSA and reported) where the CC pair in qtip operator is mutated.

This experiment has been performed and is reported in the new publication of the DPO-VqmA_Phage_ crystal structure (Gu *et al.* 2021). This paper was published while our manuscript was under review. In it, the authors used an *in vitro* DNA-binding assay to investigate the ability of VqmA_Phage_ to bind mutated P*qtip* promoters. The GG-N_9_-CC palindrome was mutated to AA-N_9_-CC or to GG-N_9_-TT. In both instances, VqmA_Phage_ maintained binding to each mutant promoter, albeit with reduced affinity. We interpret these data to mean that VqmA_Phage_ does not rely on the extended palindrome for binding, thus eliminating the referee’s second possibility that VqmA_Phage_ exclusively reads GG-N_9_-CC. Rather, the divergent core 6 nucleotides that differ between P*vqmR* and P*qtip* and that are the focus of our study, are key.

Finally, protein plasticity is harder to analyze but the authors can exploit and/or re-analyze the results presented in the manuscript using as guide the three-dimensional structures available to observe whether some of the mutations they find could confer more plasticity or alternatively induce a greater separation of the DBD at VqmA-phage than would be required to read GGN8CC. This could explain the effect observed for mutations in residues in the 201-211 region.

This is an interesting possibility, however structural information regarding VqmA_Phage_-P*qtip* or extensive modeling would be needed to test it. To address the referee’s suggestions, we now added sentences following lines 408 in the Discussion:

“Thus, while the VqmA_Phage_ residues that we identified as important for P*qtip* recognition (G201, A202, E207, and M211) do not function perfectly analogously to those in cI_lambda_ because they do not make contact with the DNA backbone, their role in site-specific recognition could be similar. A caveat of our interpretation is that, as noted, we do not have a structure of VqmA_Phage_ bound to P*qtip* and we mapped the residues identified in our VqmA_Phage_ mutagenesis to the DPO-VqmA_Vc­_-P*vqmR* crystal structure. Therefore, it remains possible that the residues we identified here do indeed make contacts with DNA. A further possibility is that the residues we identified foster increased plasticity to the VqmA_Phage_ DBDs, perhaps, allowing VqmA_Phage_ to bind the longer palindrome that exists in P*qtip*, which we discuss below. The recently reported DPO-VqmA_Phage_ crystal structure (Gu *et al.* 2021), together with the existing DPO-VqmA_Vc_ structures, could enable modeling to predict the roles played by particular residues in conferring a relaxed conformation to the VqmA_Phage_ DBDs. To our knowledge, no region analogous to the one we discovered in VqmA_Phage_ has been shown to confer promoter specificity to a transcription factor. Going forward, determining the structure of VqmA_Phage_ bound to P*qtip* DNA should reveal the mechanism enabling recognition of P*qtip* and the role that these residues play, individually and collectively, in driving DNA-binding specificity.”

It should be noted that the structure of VqmA-phage has recently been published (PDB 7DWM; Gu et al, BBRC 2021, PMID: 33895551). Although this structure should not have been available when the paper was written, I believe it may now help to understand the asymmetry.

Indeed, the availability of the structure and the companion experiments greatly aided us in our analyses, allowed us to deepen and expand our interpretation of the results in the present work, and will help us in our research going forward. In the revised manuscript, we repeatedly refer to the work by Gu *et al.* (2021) as outlined throughout this response document.

Overall, I consider that the manuscript does not meet the requirements of PLoS Genetics (“substantial evidence for its conclusions”) as the data presented are not able to determine the molecular basis of the asymmetry observed in the VqmA receptor and therefore, unfortunately, I cannot support its publication.

We hope that all of the changes we have made, the additional experiments provided in the three new figures, the expanded explanations we have included, and our references to and analyses of the newly reported structure that verifies our findings and allows us to extend our interpretations, have changed the referee’s mind on this opinion.

**REFERENCES**

1. Gu Y, Zhi SX, Yang N, Yang WS. Understanding the mechanism of asymmetric gene regulation determined by the VqmA of vibriophage. *Biochem Biophys Res Commun*. 2021; 558: 51–6.
2. Wu H, Li M, Guo H, Zhou H, Li B, Xu Q, *et al.* Crystal structure of the *Vibrio cholerae* VqmA–ligand–DNA complex provides insight into ligand-binding mechanisms relevant for drug design. *J Biol Chem*. 2019; 294(8): 2580–92.
3. Wu H, Li M, Peng C, Yin Y, Guo H, Wang W, *et al.* Large conformation shifts of *Vibrio cholerae* VqmA dimer in the absence of target DNA provide insight into DNA-binding mechanisms of LuxR-type receptors. *Biochem Biophys Res Commun*. 2019; 520(2): 399–405.
4. Huang X, Duddy OP, Silpe JE, Paczkowski JE, Cong J, Henke BR, *et al.* Mechanism underlying autoinducer recognition in the *Vibrio cholerae* DPO-VqmA quorum-sensing pathway. *J Biol Chem*. 2020; 295(10): 2916–31.
5. Papenfort K, Förstner KU, Cong JP, Sharma CM, Bassler BL. Differential RNA-seq of *Vibrio cholerae* identifies the VqmR small RNA as a regulator of biofilm formation. *Proc Natl Acad Sci U S A*. 2015; 112(7): E766–75.
6. Silpe JE, Bassler BL. A host-produced quorum-sensing autoinducer controls a phage lysis-lysogeny decision. *Cell*. 2019; 176(1-2): 268-280.e13.
7. Papenfort K, Silpe JE, Schramma KR, Cong JP, Seyedsayamdost MR, Bassler BL. A *Vibrio cholerae* autoinducer-receptor pair that controls biofilm formation. *Nat Chem Biol*. 2017; 13(5): 551–7.
